# Stomatal properties of Arabidopsis cauline and rice flag leaves and their contributions to seed production and grain yield

**DOI:** 10.1093/jxb/erac492

**Published:** 2022-12-15

**Authors:** Ming Ding, Yiyong Zhu, Toshinori Kinoshita

**Affiliations:** Plant Physiology laboratory, Graduate School of Science, Nagoya University, Nagoya 464-8602, Japan; College of Resource and Environment Science, Nanjing Agricultural University, Nanjing 210095, China; Plant Physiology laboratory, Graduate School of Science, Nagoya University, Nagoya 464-8602, Japan; Institute of Transformative Bio-Molecules (WPI-ITbM), Nagoya University, Nagoya 464-8602, Japan; University of Essex, UK

**Keywords:** Arabidopsis, cauline leaf, flag leaf, grain yield, photosynthesis, plasma membrane H^+^-ATPase, rice, stomata

## Abstract

Cauline leaves on the inflorescence stem of *Arabidopsis thaliana* may play important roles in supplying photosynthetic products to sinks, such as floral organs. Flag leaves in rice (*Oryza sativa*) have a higher photosynthetic capacity than other leaves, and are crucial for increasing grain yield. However, the detailed properties of stomata in cauline and flag leaves have not been investigated. In Arabidopsis, stomatal conductance and CO_2_ assimilation rate were higher in cauline leaves under white light than in rosette leaves, consistent with higher levels of plasma membrane (PM) H^+^-ATPase, a key enzyme for stomatal opening, in guard cells. Moreover, removal of cauline leaves significantly reduced the shoot biomass by approximately 20% and seed production by approximately 46%. In rice, higher stomatal density, stomatal conductance, and CO_2_ assimilation rate were observed in flag leaves than in fully expanded second leaves. Removal of the flag leaves significantly reduced grain yield by approximately 49%. Taken together, these results show that cauline and flag leaves have important roles in seed production and grain yield through enhanced stomatal conductance and CO_2_ assimilation rate.

## Teaser

Arabidopsis cauline leaves and rice flag leaves have important roles in seed production and grain yield, through enhanced stomatal conductance and CO_2_assimilation rate caused by increased plasma membrane H^+^-ATPase activity.

## Introduction

Stomata are small pores on the epidermis of leaves or stems that regulate gas exchange for various plant physiological processes, such as water transpiration and CO_2_ fixation by photosynthesis (Lange *et al.*, 1971; Buckley, 2005; Shimazaki *et al.*, 2007). Guard cells have evolved into two shapes: a dumbbell or a kidney (Willmer and Fricker, 1996). The dumbbell-shaped guard cells of grasses are more evolutionarily advanced than their kidney-shaped counterparts because they require fewer solutes and less water in order to open (Palevitz, 1981). Gas exchange in leaves is affected by stomatal movement and stomatal density (Harrison *et al.*, 2020). Stomatal density is generally not variable in developed leaves. In contrast, stomatal aperture has an instantaneous impact on stomatal conductance (Meidner, 1987). Environmental signals, such as light intensity, the ambient atmospheric CO_2_ concentration, and some plant hormones, control stomatal development and aperture (Hetherington and Woodward, 2003).

Blue light-induced stomatal opening is a classic adjustment of daily stomatal movement mediated by photoreceptors known as phototropins, which subsequently induce the phosphorylation of the penultimate residue, threonine (Thr), of plasma membrane (PM) H^+^-ATPase in guard cells (Kinoshita and Shimazaki, 1999; Kinoshita *et al.*, 2001). Furthermore, red light–induced stomatal opening is inhibited by the photosynthetic electron transport inhibitor 3-(3,4-dichlorophenyl)-1,1-dimethylurea (DCMU; Sharkey and Raschke, 1981; Doi and Shimazaki, 2008; Mott *et al.*, 2008; Wang *et al.*, 2011; Suetsugu *et al.*, 2014). In intact leaves, red light also induces phosphorylation of the penultimate Thr residue of PM H^+^-ATPase in guard cells (Ando and Kinoshita, 2018). This activation of PM H^+^-ATPase in guard cells pumps H^+^ out of the cell and generates an inside-out negative electrochemical potential for K^+^ uptake. The accumulation of K^+^ in the vacuole increases osmotic potential and causes water uptake into guard cells, which in turn results in swelling of the guard cells and stomatal opening (Shimazaki *et al.*, 2007).

PM H^+^-ATPase is highly conserved in plants, with multiple isoforms in various species, playing a pivotal role in plant physiology (Palmgren, 2001; Arango *et al.*, 2003; Pedersen *et al.*, 2007; Haruta *et al.*, 2015; Falhof *et al.*, 2016). PM H^+^-ATPase is commonly composed of 10 transmembrane domains, with a catalytic domain localized between the fourth and fifth transmembrane domains and its N- and C-termini in the cytoplasm (Palmgren, 2001). Post-translational modification of PM H^+^-ATPase plays an important role in light-induced stomatal opening (Kinoshita and Shimazaki, 1999; Inoue and Kinoshita, 2017). Phosphorylation of the penultimate Thr residue of PM H^+^-ATPase can activate H^+^-pumping activity by creating a binding site for 14-3-3 proteins, thus releasing the autoinhibitory C-terminus (Jahn *et al.*, 1997; Olsson *et al.*, 1998; Fuglsang *et al.*, 1999; Kinoshita and Shimazaki, 1999; Kinoshita and Shimazaki, 2002). Moreover, PM H^+^-ATPase activity is also regulated at the transcriptional and translational levels (Sze *et al.*, 1999). In general, up-regulation of PM H^+^-ATPase gene expression at the transcriptional level or enhanced synthesis of this enzyme at the translational level, generates a higher steady-state H^+^-ATPase enzyme concentration.

Up-regulated PM H^+^-ATPase gene expression (*AHA2* in Arabidopsis and poplar) in epidermal tissues increased the guard cell PM H^+^-ATPase protein level by more than 50%, and thus enhanced stomatal opening, photosynthetic activity, and plant growth (Wang *et al.*, 2014; Toh *et al.*, 2021). Furthermore, up-regulated PM H^+^-ATPase gene expression (*OSA1*) in whole rice plants increases the total PM H^+^-ATPase protein content by ~40%, leading to enhanced stomatal opening, photosynthetic activity, nutrient uptake in roots, and plant growth, with a 33% increase in grain yield even in the field (Zhang *et al.*, 2021). These results clearly indicate that PM H^+^-ATPase plays an important role in regulating stomatal movement in Arabidopsis and rice plants, thus affecting photosynthesis and even yield. Furthermore, photoperiodic flowering components affect light-induced stomatal opening in Arabidopsis (Kinoshita *et al.*, 2011; Ando *et al.*, 2013). Specifically, *SUPPRESSOR OF OVEREXPRESSION OF CO1* (*SOC1*) has been suggested to induce PM H^+^-ATPase isoforms in guard cells (Kimura *et al.*, 2015; Aoki *et al.*, 2019).

Arabidopsis and rice are model plant species for dicots and monocots, respectively. Guard cells are kidney-shaped in Arabidopsis and dumbbell-shaped in rice (Ziegler, 1987). Although, the properties and contributions to photosynthesis by guard cells in the leaves of Arabidopsis and rice have been well documented in previous studies (Wang *et al.*, 2014; Toda *et al.*, 2016), little research has focused on studying the differences in stomatal movement between leaves appearing at the vegetative stage and reproductive stage. Leaves sprouted at the reproductive stage are called cauline leaves in Arabidopsis and flag leaves in rice. Cauline leaves, which are aerial leaves attached directly to the inflorescence stem without a petiole, are formed from the shoot apical meristem during the transition to the reproductive phase (Yang and Jiao, 2016). Cauline leaves are involved in active axillary bud growth (Hempel and Feldman, 1994). They also play crucial roles in protecting flowering buds at the initial stages of the elongation of flowering stems (Aryal *et al.*, 2018), and have important roles in plant defence against bacterial infection (Patharkar *et al.*, 2017). In addition, cauline leaves are associated with a drought response, and drought-adapted Arabidopsis plants drop their cauline leaves after rewatering (Patharkar and Walker, 2016). In *Brassica napus*, cauline leaves appear after the initiation of flowering, and contribute to the provision of more carbon to seeds compared with rosette leaves (Major and Charnetski, 1976; Kirkegaard *et al.*, 2012). Note that cauline leaves showed a higher stomatal index compared with rosette leaves in Arabidopsis (Haus *et al.*, 2018). However, limited information is available regarding stomatal properties and contributions to CO_2_ assimilation, as well as seed production, in Arabidopsis cauline leaves.

Rice is an essential and universally grown crop, feeding more than half of the world’s population (Sasaki, 2005). The flag leaf is the most important source tissue in the reproductive stage of rice, and it has higher photosynthetic activity than other leaves, which contributes 50% of assimilates for grain filling (Yoshida, 1981; Evans, 1983; Ishii, 1993; Nakano *et al.*, 1995; Zhang *et al.*, 2005; Carmo-Silva *et al.*, 2017). In fact, cutting the flag leaf in rice reduces the grain yield by more than 18% (Abou-Khalifa *et al.*, 2008; Fatima *et al.*, 2019). Photosynthetic activity of the flag leaf is usually correlated with stomatal conductance, chlorophyll content, and ribulose-1,5-bisphosphate carboxylase (RuBisCO) abundance and activity (Hall *et al.*, 1978; Fischer *et al.*, 1998; Driever *et al.*, 2017). In addition, using fungicides to extend the life of the flag leaf provides continuous resources for grain filling, thereby increasing grain yields (Pepler *et al.*, 2005). However, the detailed properties of flag leaf stomata, including stomatal size, density, index values, and light-induced stomatal opening, are poorly understood.

In this study, we investigated the stomatal properties of Arabidopsis cauline leaves and rice flag leaves and their contributions to seed production and grain yields. The results show that guard cell PM H^+^-ATPase plays an important role in seed production in cauline leaves in Arabidopsis, and in grain yield in the flag leaf in rice, by widening stomatal apertures and increasing photosynthetic activity.

## Materials and methods

### Plant materials and growth conditions

*Arabidopsis thaliana* (Col-0) and *Oryza sativa* L. ssp. *japonica* ‘Nipponbare’ were used in all experiments as wild-type (WT) plants. The *A*. *thaliana* mutant *aha1-9* (*At2g18960*: SAIL_1285_D12) was obtained from the Arabidopsis Biological Resource Center (ABRC; The Ohio State University, Columbus, OH, USA). Rice mutant *osa7* (*TOS17* line NE1507) was obtained from the rice *TOS17* retrotransposon mutant panel (Toda *et al.*, 2016). The *AHA2* overexpression line under the control of the *GC1* promoter (*GC1::AHA2*) and empty vector-transformed line (*EV*) were generated in a previous study (Wang *et al.*, 2014). Arabidopsis plants used in experiments for stomatal observation, gas exchange, and immunodetection were grown hydroponically in half-strength Hoagland nutrient solution at pH 5.7–5.8 under a 16 h fluorescent light (120 µmol m^–2^ s^–1^)/8 h dark cycle at 24 °C at a relative humidity of 55–70% in a growth room, as described previously (Zeng *et al.*, 2018). The solution in the containers was changed every 2 d. The Arabidopsis plants used to measure biomass productivity, in the qRT–PCR assay, and for measurement of the nitrogen and Rubisco contents were grown in soil in the same growth room.

Rice seeds were sterilized in 10% (v/v) H_2_O_2_ for 30 min and incubated in 0.5 mM CaSO_4_ solution in the dark for 2 d. Subsequently, all seeds were spread on plastic nets floating on 1 mM CaCl_2_ solution for ~1 week in a growth chamber (NC-410HC; Nippon Medical & Chemical Instruments, Osaka, Japan) under ~150 µmol m^–2^ s^–1^ fluorescent light at 25 °C/24 °C (12 h light/12 h dark) and relative humidity of 60–80%. This was followed by the application of half-strength Kimura B nutrient solution (0.18 mM [NH_4_]_2_SO_4_, 0.09 mM KNO_3_, 0.09 mM KH_2_PO_4_, 0.18 mM Ca[NO_3_]_2_.4H_2_O, 0.27 mM MgSO_4_.7H_2_O, 6.7 µM MnCl_2_.4H_2_O, 0.015 µM [NH_4_]_6_Mo_7_O_24_.4H_2_O, 9.4 µM H_3_BO_4_, 0.15 µM ZnSO_4_.7H_2_O, 0.16 µM CuSO_4_.5H_2_O, 20 µM EDTA-Fe) at pH 5.5. The solution in the containers was replaced every 3 d.

### Observation of stomata

Stomatal apertures were measured according to the method described previously (Akiyama *et al.*, 2022) with some modifications. Epidermal tissue from cauline and rosette leaves was isolated from overnight dark-adapted 6-week-old Arabidopsis plants at zeitgeber time 3.5–4. For the detection of stomatal responses to light and abscisic acid (ABA) treatment, epidermal tissue was incubated in stomatal opening buffer (5 mM MES·bis-Tris propane, pH 6.5, 50 mM KCl, and 0.1 mM CaCl_2_) under blue light at 10 µmol m^–2^ s^–1^ and under red light (LED-R; EYELA) at 50 µmol m^–2^ s^–1^ in the presence or absence of 20 µM ABA, at 24 °C for 2.5 h. Stomatal apertures (width) are presented as means of at least 60 stomata with the standard deviation (SD). The stomatal size (long axis of each stoma), density (number of stomata per unit area), and index (number of stomata/total number of epidermal cells × 100) of Arabidopsis were measured with ImageJ (NIH, Bethesda, MD, USA) on the leaf abaxial surface with an inverted microscope system (BX43; Olympus, Tokyo, Japan). Stomatal size is presented as means ±SD of more than 120 stomata. The stomatal density and index are presented as means ±SD of at least 24 leaves. 

Stomatal size and density of rice leaves were calculated after staining with ruthenium red (Sigma-Aldrich, St. Louis, MO, USA) as described previously (Shtein *et al.*, 2017) with some modifications. Briefly, leaves of 10-day-old rice plants were excised, immediately transferred to 95% (v/v) ethanol at 4 °C for 48 h, and broken in a blender with 70% ethanol. Large fragments were incubated in Milli-Q water (MQ; Millipore, Billerica, MA, USA) containing 0.02% (v/v) Tween 20 for 2 h. Epidermal tissue was then stained with 0.01% (w/v) aqueous ruthenium red for 30 min. The samples were observed and photographed with an inverted microscope system (BX43; Olympus; Japan). The stomatal size (length of guard cells) of rice is presented as means ±SD of 420 stomata, while the stomatal density is presented as means ±SD of 15 leaves.

### Gas exchange measurements

Gas exchange measurements were performed with an LI-6400 system (LI-Cor, Lincoln, NE, USA). The flow rate, leaf temperature, and relative humidity were kept constant at 400 µmol s^–1^, 24 °C, and 40–50%, respectively, for the detection of photosynthetic rate and stomatal conductance in Arabidopsis plants. After initial adaptation to the dark for 20 min, white light (up to 700 µmol m^–2^ s^–1^) was supplied using a fibre optic illuminator with a halogen lamp (MHAB-150 W; Moritex, San Jose, CA, USA). Values are given as means ±SD of three biological replicates. For A-Ci curve measurement, the air flow rate, leaf temperature, and relative humidity were kept at 300 µmol m^–2^ s^–1^_,_ 25 °C, and 40–50%, respectively. Seven-week-old cauline and rosette leaves were initially acclimated at 400 μl l^–1^ CO_2_ under saturating white light conditions (750 µmol m^–2^ s^–1^) for 40 min and then incremented from 50 to 1000 μl l^1^ CO_2_ every 20 min. The mean value of three independent measurements with the SD was used.

The A-Ci curve was fitted for *V*_*cmax*_ (the apparent maximum of Rubisco carboxylation rate), *J* (regeneration rate of ribulose-1,5-biphosphate, also expressed as electron transport rate) and *TUP* (maximum rate of triose phosphate use) using the Excel fitting tool (Sharkey *et al.*, 2007; Walker *et al.*, 2013). Stomatal conductance was obtained from the data points collected at 400 μl l^–1^ CO_2_. The mean represents value of three independent measurements with the SD.

For rice, the flow rate, leaf temperature, and relative humidity were kept constant at 500 µmol m^–2^ s^–1^, 24 °C, and 60–70%, respectively. Leaves were measured under photosynthetically saturating white light (up to 1000 µmol m^–2^ s^–1^). The mean value of three independent measurements with the SD was used.

### Immunodetection of plasma membrane H ^*+*^ -ATPase

Immunoblotting analyses were performed according to the method described previously (Hayashi *et al.*, 2010) with minor modifications. For Arabidopsis, 0.1 g of cauline leaves from 6-week-old Col-0 and *aha1-9* mutant plants were immediately homogenized in liquid N_2_ with a mortar and pestle, and then resuspended in 200 µl of ice-cold homogenization buffer (50 mM MOPS-KOH, pH 7.5, 5 mM EDTA, 100 mM NaCl, 0.5 mM PMSF, 10 µM leupeptin, 2 mM DTT, 10 mM NaF, and 0.5 mM ammonium molybdate). Next, 200 µl of SDS solubilization buffer was added to the extracts of samples followed by centrifugation at 10 000 × *g* for 1 min at 24 °C The supernatant was isolated for (SDS-PAGE and immunoblotting. PM H^+^-ATPase protein was detected with anti-Arabidopsis H^+^-ATPase antibodies from rabbit. For rice plants, 0.1 g of samples of flag leaves from 49-day-old WT and *osa7* mutants were used for immunoblotting as described above, except that the antibody used was anti-rice H^+^-ATPase antibodies from rabbit. Relative PM H^+^-ATPase levels were estimated by assessing the signal intensity with Image J (NIH, Bethesda, MD, USA). Mean values of three independent measurements with the SD were used.

Anti-rice H^+^-ATPase antibodies, specific for the conserved catalytic domain of the PM H^+^-ATPase of rice (*OSA7*, LOC_Os04g56160), were raised in rabbits. We used the primers 5ʹ-GCCGGATCCGTTCTGTGCAGTGACAAGA-3ʹ and 5ʹ-GCCGAAGCTTTCATCCAAGCCTCCTACCA-3ʹ to amplify the *OSA7* region from first-strand cDNA synthesized using RNA from rice leaves by PCR. The resulting amplified PCR product of 973–1578 bp of *OSA7*, containing a *Bam*HI site at the 5ʹ-terminus and a *Hin*dIII site at the 3ʹ-terminus, was cloned into the *Bam*HI and *Hin*dIII sites of the pET30a vector (Merck, Darmstadt, Germany). The purified proteins from *Escherichia coli* (BL21) were used as an antigen.

Immunohistochemical detection of PM H^+^-ATPase in guard cells was performed as described previously (Hayashi *et al.*, 2011). Firstly, epidermal tissue from cauline leaves and rosette leaves from 6-week-old Col-0, *aha1-9* mutant, and *GC1::AHA2*-ox plants was fixed using 4% (w/v) formaldehyde with 0.1% (w/w) glutaraldehyde in microtubule stabilization buffer (MTSB) at 4 °C in the dark for 2 h. After washing with phosphate-buffered saline (PBS), the epidermal tissue was incubated for 10 min with pure methanol at 37 °C to remove chlorophyll, and mounted on MAS adhesive-coated microscope slides (Matsunami Glass, Bellingham, WA, USA). The tissue was then digested with a supernatant of 3% (w/v) Driselase (Sigma-Aldrich) and 0.5% (w/v) Macerozyme R-10 in PBS at 37 °C for 45 min. After digestion, the slides were washed three times with PBS, for 5 min each time. The epidermis was made permeable with 3% (v/v) IGEPAL CA-630 (MP Biomedicals, Irvine, CA, USA) and 10% (v/v) DMSO in PBS 24 °C for 30 min. After washing, the samples were incubated with blocking solution consisting of 3% (w/v) bovine serum albumin (BSA) fraction V (Gibco-BRL, Gaithersburg, MD, USA) in PBS at 24 °C for 1 h. Primary antiserum (anti-Arabidopsis H^+^-ATPase) was then applied at a dilution of 1:3000 in blocking solution and incubated overnight at 4 °C. Secondary antibody (Alexa Fluor 488-conjugated goat anti-rabbit IgG; A11034; Invitrogen, Carlsbad, CA, USA) was applied at a dilution of 1:500 in blocking solution at 37 °C for 3 h. Lastly, the specimens were covered with a cover glass and 50% (v/v) glycerol for further analysis.

### Estimation of plasma membrane H ^*+*^ -ATPase in Arabidopsis guard cells

The levels of PM H^+^-ATPase in guard cells from Arabidopsis rosette and cauline leaves were determined by assessing the Alexa Fluor 488 fluorescence intensity with ImageJ (NIH, Bethesda, MD, USA). The value for each specimen was calculated as the geometric mean of more than 30 independent measurements of pairs of guard cells from three to four leaves. Mean values from at least three independent biological replicates with SD were used.

### qRT–PCR analysis

Quantitative reverse transcription-PCR (qRT–PCR) analysis was performed according to the method described in Ando *et al.* (2013). In the first step, total RNA was isolated from guard cell-enriched epidermal fragments of leaves from 4-week-old Col-0 or leaves from *GC1::AHA2* plants using an RNeasy Plant Mini Kit (Qiagen, Germantown, MD, USA). First-strand cDNA was synthesized from total RNA with a PrimeScript II First Strand cDNA Synthesis Kit using oligo(dT) primers (Takara, Kyoto, Japan) followed by qRT–PCR with a StepOne Real-Time PCR system (Applied Biosystems, Foster City, CA, USA) and Power SYBR Green PCR Master Mix. Gene expression was assessed relative to β-tubulin and ubiquitin 5 using the comparative cycle threshold (^ΔΔ^Ct) method (Livak and Schmittgen, 2001). The primers used are as follows: for *AHA2*, 5ʹ-TTGTTGAACGTCCTGGAGCA-3ʹand 5ʹ-AATTCCCAGTTGGCGTAAACC-3ʹ; for *SOC1*, 5ʹ-ATAGGAACATGCTCAATCGAGGAGCTG-3ʹ and 5ʹ-TTTCTTGAAGAACAAGGTAACCCAATGC-3ʹ; for *β*-*TUB2*, 5ʹ-AAACTCACTACCCCCAGCTTTG-3ʹ and 5ʹ-CACCAGACATAGTAGCAGAAATCAAGT-3ʹ; and for *UBQ5*, 5ʹ-ACCACTTCGACCGCCACTACT-3ʹ and 5ʹ-ACGCCTAAGCCTGCTGGTT-3ʹ. Mean values of three biological replicates with the SD were used.

### Leaf removal experiments in Arabidopsis and rice

Cauline leaf removal (CLR) experiments were performed as follows: Arabidopsis plants were grown in soil for ~5 weeks until bolting. After removing the first cauline leaf, all new cauline leaves were removed every day until maturity. Control plants were grown under the same conditions without leaf removal. After 6 weeks, the fresh weight of the shoots, average silique numbers, and average silique dry weight were calculated. The mean values of six independent measurements with the SD were used for further analysis.

Flag leaf removal (FLR) experiments were performed as follows. Rice plants were grown hydroponically in a growth chamber, and the flag leaf was removed at the collar formation (FLR-R2), panicle exertion (FLR-R3), and anthesis (FLR-R4) stages. Control rice plants were grown under the same conditions without leaf removal.

### Rice yield experimental design

For the yield experiments, 16 WT plants and four *osa7* mutant 1-week-old plants grown in 1 mM CaCl_2_ were selected and cultivated in four 3.7 l containers with half-strength Kimura B nutrient solution at pH 5.5. Each container contained four WT seedlings and one *osa7* mutant kept in holes in extruded polystyrene plates floating on the nutrient solution. The flag leaves of three WT plants in each container were plucked at different growth stages (R2, R3, and R4). All plants were grown in a growth chamber under the same growth conditions until maturity. During that time, the treatments and plants were completely randomized by rearranging the container position every 3 d. The mean of four independent measurements with the SD for agronomic traits and grain yields were used.

### Measurement of chlorophyll content

The chlorophyll content of rice leaves was determined as described previously (Sartory and Grobbelaar, 1984; Xiong *et al.*, 2015). To extract chlorophyll, a leaf sample (0.1 g) was incubated in a buffer containing 90% ethanol (v/v) for 12 h at 4 °C in darkness. A UH5300 Spectrophotometer (Hitachi, Tokyo, Japan) was used to determine the absorbance of the chlorophyll extract at wavelengths of 645 nm and 663 nm. The mean value of three biological replicates with the SD was used.

### SDS-PAGE for Rubisco content determination

Fresh leaves of 49-day-old WT plants were used for protein extraction and subsequent detection using SDS-PAGE. Leaf tissues (4 mg) were homogenized in 100 µl of extraction buffer (pH 6.8) containing 100 mM Tris-HCl, 2% (w/v) SDS, 1 mM EDTA, 1 mM PMSF, 20 µM leupeptin, 50 mM DTT, 2.5 mM NaF, 20% (v/v) glycerol, and 0.012% Coomassie brilliant blue (CBB), followed by centrifugation at 14 000 × *g* for 5 min at 24 °C. Next, 20 µl of supernatant was used for SDS-PAGE and electrophoresed for 80 min at 100 V, followed by placing the gel in fixation solution [50% (v/v) methanol and 10% (v/v) acetic acid] for 15 min and staining in CBB solution [60 mg l^–1^ of CBB G-250 and 10% (v/v) acetic acid] for 10 min. Total Rubisco content, including the large and small subunits, in cauline leaves is presented relative to that in rosette leaves (set as 1). The mean value of nine independent measurements with the SD was used for further analysis.

### Determination of total nitrogen content 

Cauline and rosette leaves were harvested from 6-week-old Col-0 plants and photographed. Leaf areas were measured using ImageJ software and the recorded values were normalized to the true leaf area. Then the leaves were dried in a forced-air oven at 70 °C for ~48 h to a constant weight, for dry weight measurement. The total nitrogen (N) content was determined using a Flash2000-DELTAplus Advantage ConFloIII System (ThermoFisher Scientific, USA) at Shoko Science Co., Ltd (Kanagawa, Japan). The mean value of six independent measurements with the SD of the N content (µg) per leaf area (cm^2^) was used.

### Measurement of sucrose content

Sucrose content (mg sucrose per g fresh weight) was measured according to the method of Stitt *et al.* (1989). Samples (0.1 g) were extracted three times with 4 ml of 80% (v/v) ethanol at 80 °C for 20 min each time, and then incubated for 10 min in boiling water. The volume of the extract was brought up to 10 ml with 80% ethanol. Sucrose standard solutions were made with concentrations of 0, 10, 20, 40, and 60 g sucrose ml^–1^ in 80% ethanol. Samples of 0.5 ml extract and standard solutions were brought up to 2 ml with distilled water followed by the addition of 200 µl of 2 M NaOH and incubating at 100 °C for 5 min to remove reducing sugars. After cooling to 25 °C, 1 ml of 9% resorcinol and 5 ml of pure sulfuric acid were added slowly, and allowed to react with the extracts for 15 min at 80 °C. After cooling to 24 °C, 0.4 ml aliquots of the sucrose standard solutions and samples were used to calculate the optical density (OD) at a wavelength of 480 nm using a spectrophotometer (UH5300; Hitachi; Japan). The OD_480_ value of a 0 g ml^–1^ standard solution was set as 0 and the sucrose content was estimated based on OD_480_ values of the samples using a standard curve. The mean values of three independent measurements with the SD of sucrose content in the leaves and seeds of rice and Arabidopsis were used.

### Statistical analysis

The statistical significance of the difference between two independent means was assessed using a two-tailed Student’s *t*-test. The statistically significant differences among means were determined by a one-way ANOVA followed by Tukey’s multiple comparisons test. In all analyses, *P*<0.05 was taken to indicate statistical significance. Statistical analyses were performed with Prism 9 (GraphPad Software, La Jolla, CA, USA), and figures were drawn in PowerPoint (Microsoft Corporation, Redmond, WA, USA).

## Results

### Differences in stomatal properties of cauline and rosette leaves of Col-0 WT *Arabidopsis thaliana*

**Fig. 1.**
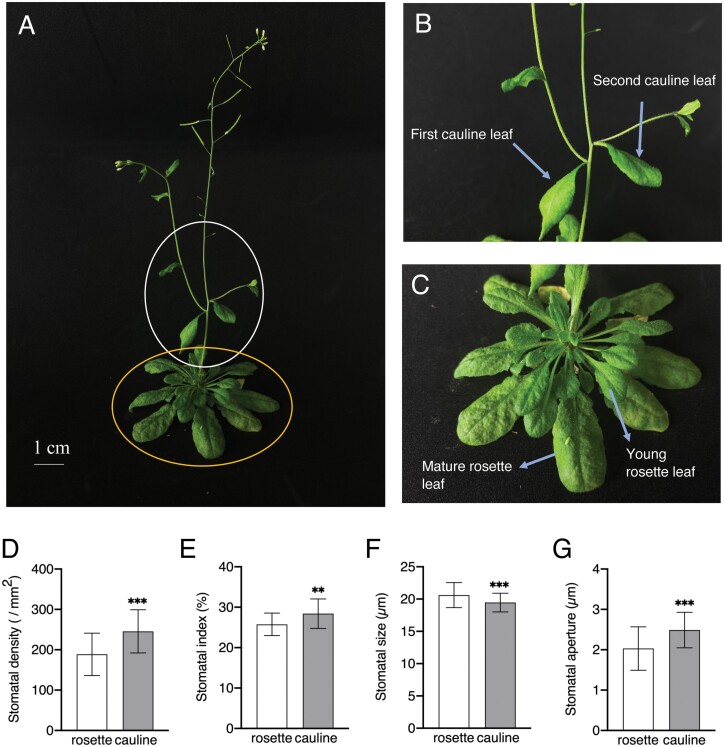
Characteristics of stomata in cauline and rosette leaves. (A) Image of 6-week-old Col-0. The white and yellow circled regions are magnified in (B) and (C), respectively. Scale bar=1 cm. (B) Phenotype of first and second cauline leaves attached to the primary inflorescence. Blue arrows indicate positions of first and second cauline leaves. (C) Phenotype of young and mature rosette leaves. Blue arrows indicate positions of young and mature rosette leaves. (D) Stomatal density of cauline and rosette leaves. Values represent means ±SD (*n*=3) of at least 10 leaves in each experiment. (E) Stomatal index of cauline and rosette leaves. Data are means ±SD (*n*=3) of at least eight leaves in each experiment. (F) Stomatal size of cauline and rosette leaves. Data are means ±SD (*n*=3) of at least 50 stomata in each experiment. (G) Stomatal apertures of cauline and rosette leaves in the light at growth stage. Data are means ±SD (*n*=3) of at least 20 stomata in each experiment. The significant differences between corresponding rosette leaves in (D)–(G) was determined with Student’s *t-*test (*P<0.05; **P<0.01; ***P<0.001).

Cauline leaves are the aerial leaves attached directly to the inflorescence stem without a petiole (Fig. 1A, B). A previous study showed that the young cauline leaf has a higher stomatal density and index than rosette leaves (Haus *et al.*, 2018). In this study, we investigated stomatal phenotypes using first cauline leaves and mature rosette leaves (Fig. 1A–C). Consistent with a previous study, the stomatal density and index of the abaxial epidermis from cauline leaves were 30% and 12% higher, respectively, than in rosette leaves in Col-0 (Fig. 1D, E). In contrast, the stomatal size of cauline leaves was slightly smaller than that of rosette leaves (Fig. 1F). Next, we investigated stomatal apertures under controlled growth conditions and found that stomata from cauline leaves opened wider than those from rosette leaves in Col-0 (Fig. 1G). To eliminate the effects of leaf position and evaluate ABA effects on stomatal opening in cauline and rosette leaves, we incubated epidermal tissue isolated from overnight dark-adapted cauline leaves or rosette leaves in stomatal opening buffer under 50 µmol m^−2^ s^−1^ red light and 10 µmol m^−2^ s^−1^ blue light for 2.5 h with or without 20 µM ABA. The stomata from cauline leaves opened significantly wider than those from rosette leaves under light conditions, but closed under ABA treatment, and showed no difference from those of rosette leaves (*P*<0.001; Supplementary Fig. S1). These results show that the position of cauline and rosette leaves is not the reason for enhanced stomatal apertures in cauline leaves, and that there is no difference in ABA sensitivity in stomata from both sources.

### The plasma membrane H ^*+*^-ATPase expression affects stomatal aperture and photosynthetic activity

**Fig. 2.**
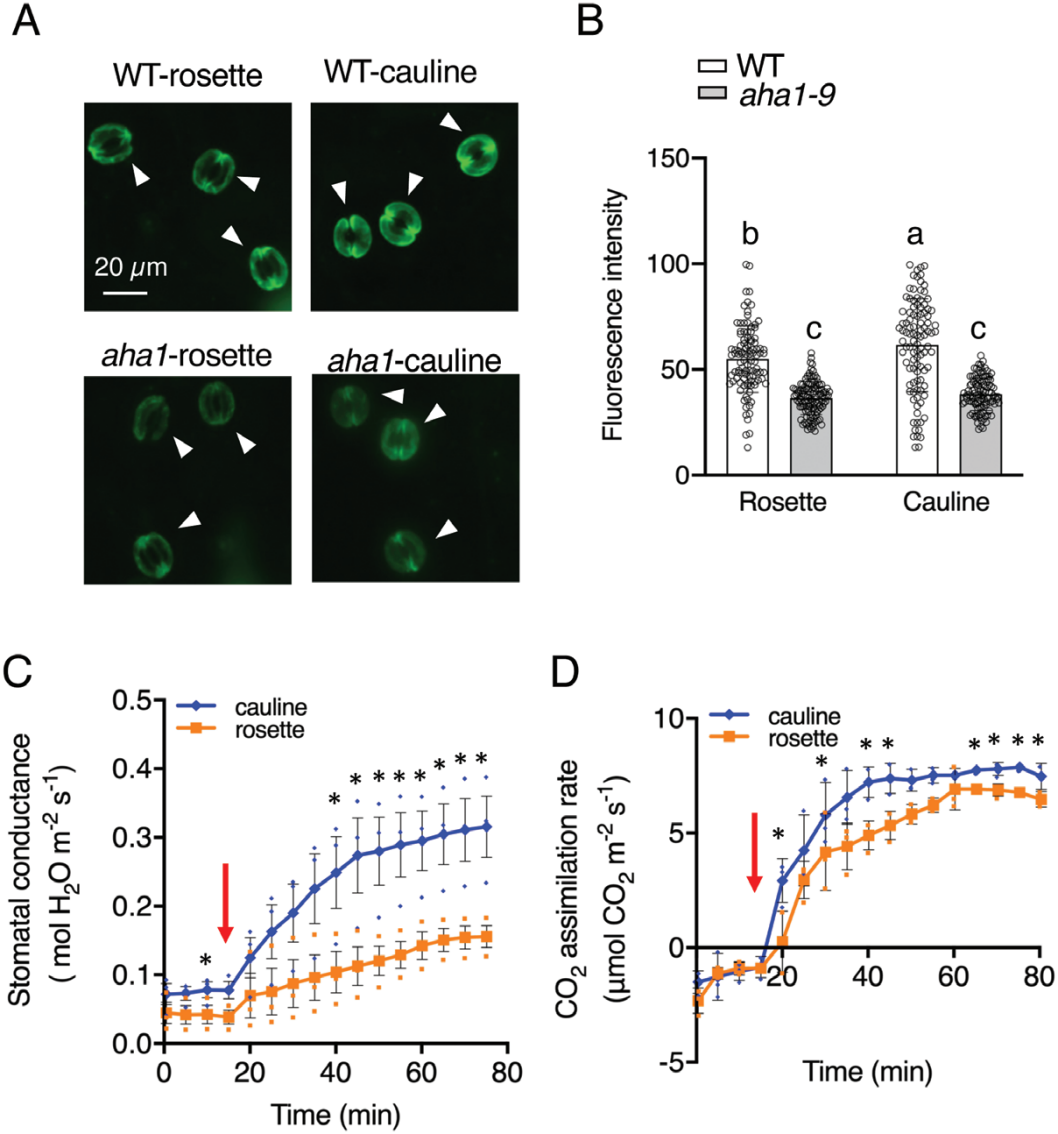
Plasma membrane H^+^-ATPase and photosynthetic properties of cauline and rosette leaves. (A) Typical fluorescence images of immunohistochemical detection of guard cell PM H^+^-ATPase in the epidermis of cauline and rosette leaves of wild-type Col-0 (WT) and the *aha1-9* mutant. Arrowheads indicate positions of stomata in the epidermis. Scale bar=20 µm. (B) Intensities of the fluorescent signals from PM H^+^-ATPase in guard cells. Data are means ±SD (*n*=3) of at least 30 stomata in each experiment. Different letters indicate significant differences at *P* <0.01 according to a one-way ANOVA with Tukey’s multiple comparisons test. (C) Stomatal conductance and (D) CO_2_ assimilation rate of cauline and rosette leaves of Col-0. Measurements were conducted under white light (700 µmol m^–2^ s^–1^). The leaf temperature was maintained at 24 °C and the relative humidity in the leaf chamber was kept at 40–50%. Data are means ±SD (*n*=3). Asterisks indicate significant differences from corresponding rosette leaves (Student’s *t* test; *P<0.05). Error bars are not shown if smaller than the symbol. Red arrows indicate the time the lights were on. Small circles in (B)–(D) indicate data points for individual experiments.

Wang *et al.* (2014) demonstrated that up-regulation of PM H^+^-ATPase gene expression in Arabidopsis guard cells increases the PM H^+^-ATPase protein expression and stomatal opening in response to light, compared with WT. Therefore, differences in stomatal aperture between cauline and rosette leaves (Fig. 1G) may be related to the guard cell PM H^+^-ATPase protein content. To test this hypothesis, we performed immunohistochemical analyses to examine guard cell PM H^+^-ATPase protein expression in the epidermis of both cauline and rosette leaves, using specific antibodies against PM H^+^-ATPase. The guard cell PM H^+^-ATPase protein expression was 12% higher in cauline leaves than in rosette leaves (Fig. 2A, B). Next, we examined stomatal conductance and photosynthetic activity (CO_2_ assimilation rate) of cauline and rosette leaves using a gas exchange system. Cauline leaves showed slightly higher stomatal conductance in the dark compared with rosette leaves. Illumination with white light at 700 µmol m^–2^ s^–1^ increased stomatal conductance in both leaf types. Stomatal conductance was saturated around 60 min after the start of light illumination in rosette leaves, and around 40 min in cauline leaves. The average stomatal conductance and photosynthetic rate in the cauline leaves were approximately 2-fold and 10% higher, respectively, than in rosette leaves ~60 min after the start of light illumination (Fig. 2C, D).

**Fig. 3.**
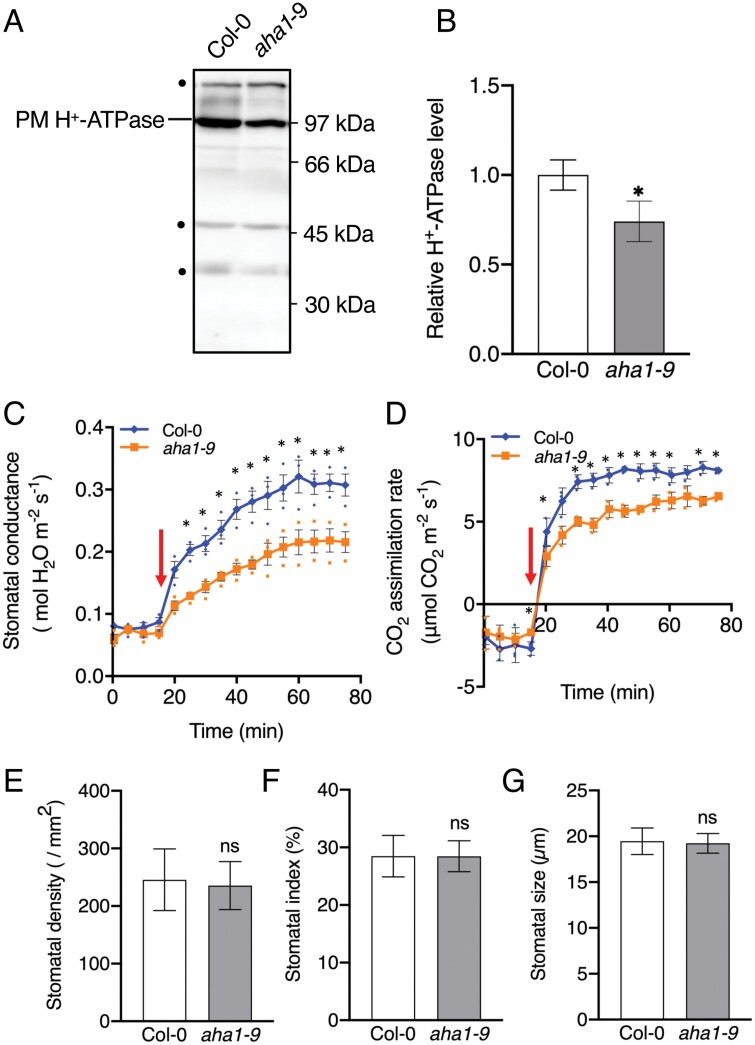
Gas exchange parameters of cauline leaves of the *aha1-9* mutant. (A) PM H^+^-ATPase expression was determined in cauline leaves of Col-0 and *aha1-9* mutants by immunoblotting protein extracts using a specific anti-Arabidopsis H^+^-ATPase antibody. The arrowhead indicates the position of PM H^+^-ATPase. Dots indicate non-specific proteins recognized by anti-Arabidopsis H^+^-ATPase. Numbers on the right represent the positions of molecular weight markers. (B) H^+^-ATPase protein expression in cauline leaves of *aha1-9* mutants relative to Col-0 given an arbitrary value of 1. Data are means ±SD (*n*=3) of three leaves in each experiment. Asterisk indicates significant difference from the corresponding Col-0 (Student’s *t*-test; *P<0.05). (C) Stomatal conductance and (D) CO_2_ assimilation rate of cauline leaves of Col-0 and *aha1-9* mutants. Small circles in (C) and (D) represent data points for individual experiments. Measurements were conducted under white light (700 µmol m^–2^ s^–1^). The leaf temperature was maintained at 24 °C and the relative humidity in the leaf chamber was kept at 40–50%. Data are means ±SD (*n*=3). Asterisks indicate significant differences from corresponding rosette leaves (Student’s *t*-test; *P<0.05). Error bars are not shown if smaller than the symbol. Red arrows indicate the time the lights were on. (E) Stomatal density in Col-0 and *aha1-9* mutant plant cauline leaves. Values represent means ±SD (*n*=3) of 9–10 leaves in each experiment. (E) Stomatal index of Col-0 and *aha1-9* mutant cauline leaves. Data are means ±SD (*n*=3) of 9–10 leaves in each experiment. (F) Stomatal size in Col-0 and *aha1-9* mutant cauline leaves. Data are means ±SD (*n*=3) for 40–50 stomata in each experiment.

To further determine the importance of PM H^+^-ATPase in stomatal opening in cauline leaves, we investigated stomatal phenotypes in the *aha1-9* mutant, a knock out of the major PM H^+^-ATPase isoform *AHA1* (Osakabe *et al.*, 2016; Yamauchi *et al.*, 2016). Western blot analyses showed that the PM H^+^-ATPase protein level in cauline leaves of the *aha1-9* mutant was decreased by 32% compared with Col-0 (Fig. 3A, B). In addition, the guard cell PM H^+^-ATPase protein level in the *aha1-9* mutant was significantly reduced in cauline leaves compared with Col-0 (*P*<0.001; Fig. 2A, B). Gas exchange experiments showed that both stomatal conductance and CO_2_ assimilation rate were significantly reduced in cauline leaves of the *aha1-9* mutant compared with Col-0 (*P*<0.05; Fig. 3C, D). Moreover, the stomatal density, index, and size in cauline leaves from Col-0 and *aha1-9* mutant plants showed no significant difference (Fig. 3E–G).

Next, we investigated stomatal phenotypes in transgenic plants overexpressing *AHA2* under the control of the *GC1* guard cell strong promoter (*GC1::AHA2*), which shows enhanced growth compared with Col-0 (Supplementary Fig. S2A; Wang *et al.*, 2014). *GC1::AHA2* showed approximately three times higher *AHA2* gene expression in guard cell-enriched epidermis from cauline leaves compared with Col-0 (Supplementary Figs S2B, S3A), which contributed to ~50% enhanced PM H^+^-ATPase protein expression in the guard cells of cauline leaves (Supplementary Fig. S2C, D). The stomatal aperture, stomatal conductance, and CO_2_ assimilation rate of cauline leaves were significantly higher in *GC1::AHA2* than in Col-0 (*P*<0.001; Supplementary Fig. S2E–G). In addition, the stomatal density, index, and size in Col-0 and *GC1::AHA2* cauline leaves showed no significant difference (*P*>0.05; Supplementary Fig. S2H–J). These results suggest that PM H^+^-ATPase plays an important role in light-induced stomatal opening in cauline leaves of Arabidopsis.

**Fig. 4.**
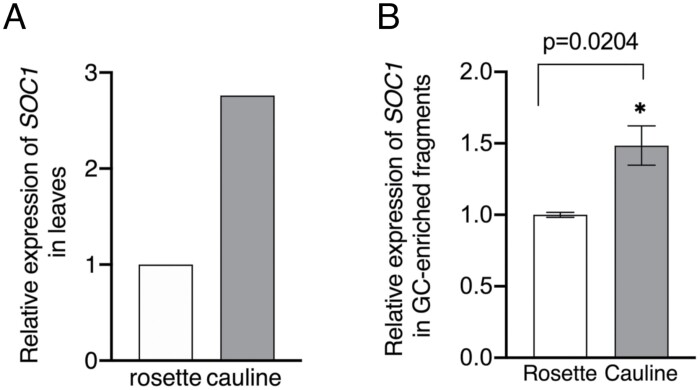
Relative expression levels of *SOC1* in rosette and cauline leaves and guard cell-enriched epidermal fragments of 6-week-old Col-0. (A) Relative expression levels of *SOC1* in rosette and cauline leaves. The expression level of *SOC1* was obtained from the Arabidopsis eFP browser (https://bar.utoronto.ca/efp/cgi-bin/efpWeb.cgi). (B) Relative expression level of *SOC1* in guard cell-enriched fragments of rosette and cauline leaves. Data are means ±SD (*n*=6). Gene expression was calculated relative to *β-TUB2.* Asterisks indicate significant differences from the corresponding rosette leaves (Student’s *t*-test; *P<0.05).

In addition, we investigated the expression level of *SOC1* as it is an important factor for the reproductive phase, and has been suggested to induce PM H^+^-ATPase expression in guard cells (Kimura *et al.*, 2015; Aoki *et al.*, 2019). First, we analysed the expression level of *SOC1* in cauline and rosette leaves using the Arabidopsis eFP Browser database (https://bar.utoronto.ca/efp/cgi-bin/efpWeb.cgi), and found that the expression level of *SOC1* in cauline leaves was approximately 2.8 times higher than that in rosette leaves (Fig. 4A). Then, we investigated the expression level of *SOC1* in guard cell-enriched epidermis in cauline and rosette leaves. The expression level of *SOC1* in guard cell-enriched epidermis from cauline leaves was ~40% higher than that from rosette leaves (Fig. 4B; Supplementary Fig. S3B).

### The physiological role of cauline leaves in seed production

**Fig. 5.**
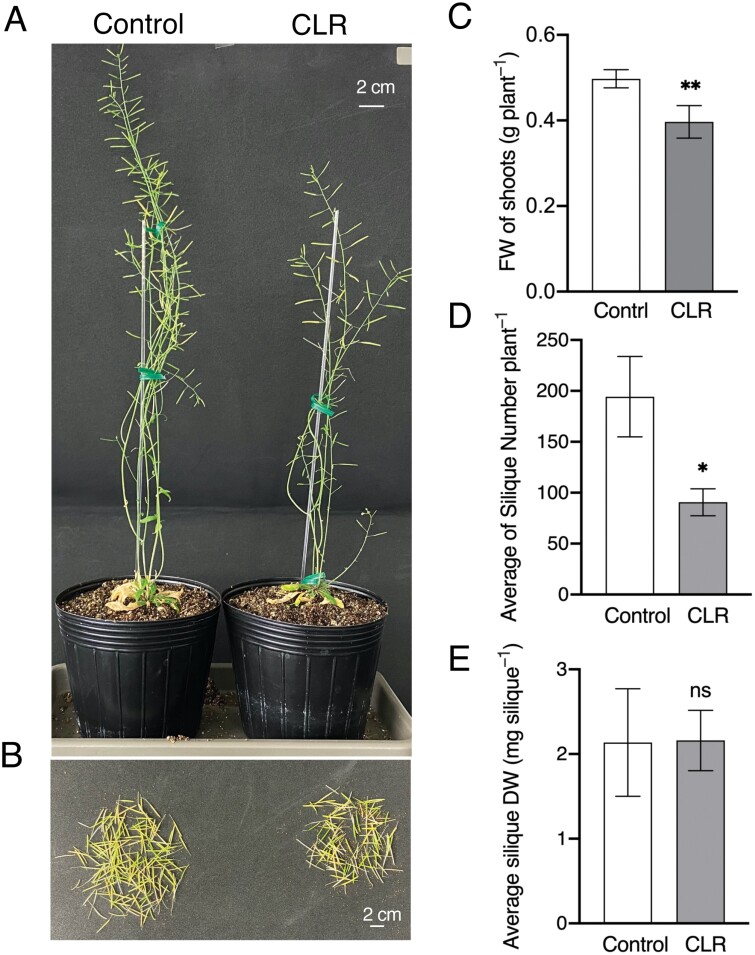
Effects of cauline leaf removal (CLR) treatment on plant growth and productivity. (A) Typical phenotypes of control (no leaf removal) and CLR plants grown in the soil under 150 µmol m^–2^ s^–1^ white light for 8 weeks in a growth room. Scale bars=2 cm. (B) Typical image of fresh siliques of one control and CLR plant. Scale bars=2 cm. (C) Above-ground fresh weights of 6-week-old control and CLR plants. Data are means ±SD (*n*=6). Asterisks indicate significant differences from corresponding control plants (Student’s *t*-test; **P<0.01). (D) Average number of siliques per plant. Data are means ±SD (*n*=6). Asterisks indicate significant differences from corresponding control plants (Student’s *t* test; *P<0.05). (E) Average silique dry weight per plant calculated as the total silique dry weight of each plant divided by the total number of siliques of each plant. Data are means ±SD (*n*=6). Differences were examined for significance with Student’s *t*-test (ns, not significant).

To investigate the importance of cauline leaves in seed production during the reproductive stage, we performed CLR treatment by cutting off cauline leaves when they appeared, then examined their biomass and seed production (Fig. 5A). The biomass of 6-week-old CLR plants was reduced by 20% compared with controls (Fig. 5C). Similarly, the number of siliques was significantly reduced in CLR plants by ~53% (*P*<0.05; Fig. 5B, D), with average silique weight comparable to controls (Fig. 5E). In addition, we found that the *aha1-9* mutant showed less seed production compared with Col-0, although the average silique weight was comparable between the mutant and WT lines (Supplementary Fig. S4), and sucrose content in cauline leaves was significantly reduced in *aha1-9* compared with Col-0 (*P*<0.01; Supplementary Fig. S5). It is possible that the reduction in photosynthetic product in cauline leaves is an important factor in the reduced silique yield in Arabidopsis. Furthermore, we investigated the effects of the removal of cauline leaves in Col-0, empty vector transformants (EV), and *AHA2*-ox; seed production was reduced by ~45.6%, 42%, and 34%, respectively, compared with the corresponding controls with no leaves removed (Supplementary Fig. S6). These results suggest that cauline leaves are important for seed production, and that PM H^+^-ATPase in guard cells is involved in seed production in Arabidopsis.

### The rice flag leaf exhibits enhanced stomatal characteristics and photosynthetic activity

**Fig. 6.**
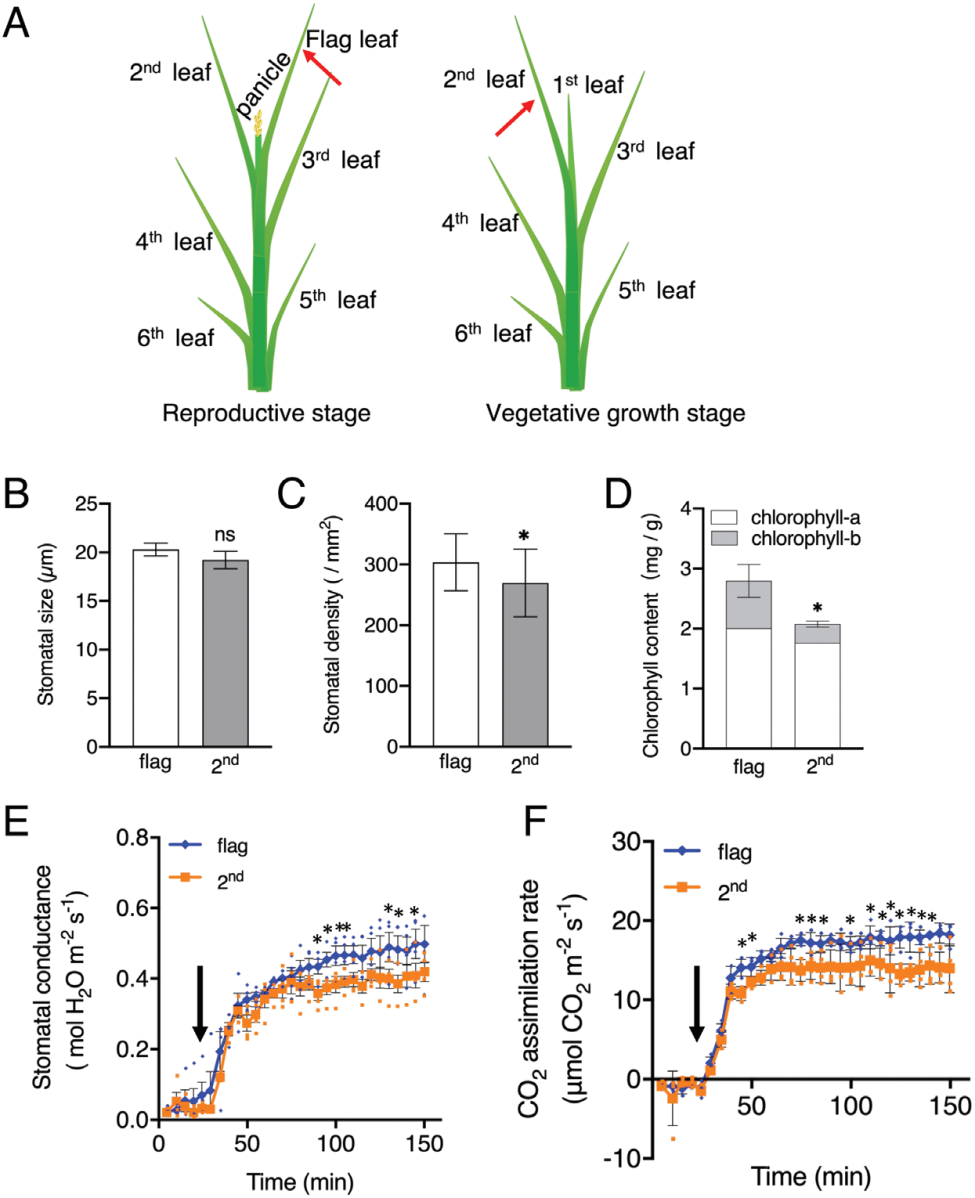
Stomatal and photosynthetic characteristics of the flag leaf of rice at the reproductive stage and fully expanded second leaf of rice at the vegetative stage. (A) Schematic representation of leaf positions of rice plants at reproductive growth stage and vegetative stage. Leaf positions are numbered from newest to oldest. Red arrows indicate the position of the flag leaf at the reproductive stage and fully expanded second leaf at the vegetative stage. (B) Stomatal size of the flag leaf and second leaf. Data are means ±SD (*n*=3) of 140 stomata in each experiment. Differences from the corresponding flag leaf were examined for significance with Student’s *t* test (ns, not significant). (C) Stomatal density of the flag leaf and second leaf. Data are means ±SD (*n*=3) of five leaves in each experiment. Asterisks indicate significant differences from the corresponding flag leaf (Student’s *t*-test; *P<0.05). (D) Chlorophyll content of the flag leaf and second leaf. Data are means ±SD (*n*=3) of four leaves in each experiment. Asterisks indicate significant differences from the corresponding flag leaf (Student’s *t*-test; *P<0.05). (E) Stomatal conductance and (F) CO_2_ assimilation rate of the flag leaf and second leaf were determined under white light of 1000 µmol m^−2^ s^−1^. The leaf temperature and relative humidity in the leaf chamber were maintained at 24 °C and 60–70%, respectively. Data are means ±SD. Small circles in (E) and (F) indicate data points for individual experiments. Experiments were repeated three times with similar results. Asterisks indicate significant differences from the corresponding second leaf (Student’s *t*-test; *P<0.05). Error bars are not shown if smaller than the symbol. Black arrows indicate the time lights were on.

The rice flag leaf is the last to emerge and is an indicator of the transition from the vegetative to the reproductive stage. Photosynthetic activity is higher in the flag leaves than in other older leaves (Evans, 1983; Zhang *et al.*, 2005; Carmo-Silva *et al.*, 2017). In previous studies, however, measurements were performed on leaves of different ages derived from the same plants. These results may have been affected by age, because the flag leaf is the youngest leaf. Therefore, we compared stomatal properties and photosynthetic activity of the flag leaf at the reproductive stage with those of the fully expanded second youngest leaf of rice at the vegetative growth stage, which is of similar age (Fig. 6A). We found no difference in stomatal size between the flag leaf and second youngest leaf (Fig. 6B). Firstly, fresh leaf blades were stained with ruthenium red and observed by microscopy (Supplementary Fig. S7). Interestingly, the stomatal size of the flag leaf was not significantly different from that of the second leaf (*P*>0.05; Fig. 6B), but the stomatal density was increased by 12% compared with the second youngest leaf (Fig. 6C). In addition, we found that the total content of chlorophyll-a and chlorophyll-b in the flag leaf was increased by ~35% compared with the second leaf (Fig. 6D). Gas exchange analysis showed that the stomatal conductance and CO_2_ assimilation rate were both higher in the flag leaf than in the second youngest leaf (Fig. 6E, F).

### The *osa7* mutant shows reduced stomatal conductance and photosynthetic activity in the flag leaf

**Fig. 7.**
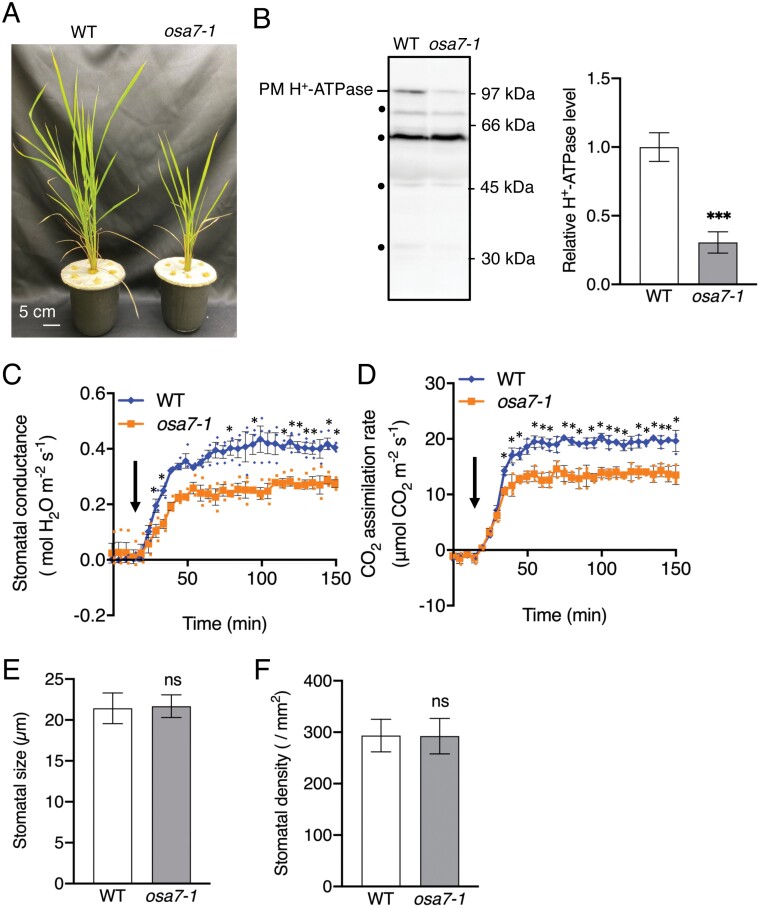
Stomatal and photosynthetic properties of *osa7-1* mutants of the flag leaf. (A) Typical phenotypes of 7-week-old WT and *osa7-1* mutants grown hydroponically in a light incubator under approximately 150 µmol m^−2^ s^−1^ fluorescent light at 25 °C/24 °C (12 h/12 h) and 60–80% relative humidity. Scale bars=5 cm. (B) PM H^+^-ATPase expression in the flag leaf of WT and *osa7-1* mutants was determined by immunoblotting analysis using a specific anti-rice H^+^-ATPase antibody. The PM H^+^-ATPase content in *osa7-1* mutant plants was normalized relative to that of WT given an arbitrary value of 1. Data are means ±SD (*n*=3). Three flag leaves were used in each experiment. Asterisks indicate significant differences from the corresponding WT (Student’s *t*-test; ***P<0.001). The arrowhead indicates the position of PM H^+^-ATPase. Dots indicate non-specific proteins recognized by anti-rice H^+^-ATPase antibodies. Numbers on the left represent the positions of molecular weight markers. (C) Stomatal conductance and (D) CO_2_ assimilation rate of the flag leaf of WT and *osa7-1* mutants under white light of 1000 µmol m^−2^ s^−1^. Small circles in (C) and (D) indicate data points for individual experiments. The leaf temperature and relative humidity in the leaf chamber were maintained at 24 °C and 60–70%, respectively. Data are means ±SD (*n*=3). Asterisks indicate significant differences from the corresponding second leaf (Student’s *t*-test; *P<0.05). Error bars are not shown if smaller than the symbol. Black arrows indicate the time lights were on. (E) Stomatal size of the flag leaf of WT and *osa7-1* mutant plants. Data are means ±SD (*n*=3) of 30 stomata in each experiment. Differences from the corresponding WT were examined for significance with Student’s *t*-test (ns, not significant). (F) Stomatal density of the flag leaf of WT and *osa7-1* mutant plants. Data are means ±SD (*n*=3) of three leaves in each experiment. Differences from the corresponding WT were examined for significance with Student’s *t-*test (ns, not significant).

*OSA7*, a major isoform of *O*. *sativa* PM H^+^-ATPase (*OSA*), is involved in light-dependent stomatal opening (Toda *et al.*, 2016). The *osa7* null mutant showed severe growth defects (Fig. 7A). Western blot analyses using anti-rice H^+^-ATPase antibodies showed that the PM H^+^-ATPase protein level was markedly reduced in flag leaves of the *osa7* mutant (Fig. 7B; Toda *et al.*, 2016). In addition, stomatal conductance and CO_2_ assimilation rate were significantly reduced in the flag leaf of the *osa7* mutant compared with WT (*P*<0.05; Fig. 7C, D). However, there were no differences in flag leaf stomatal size or density between WT and the *osa7* mutant (Fig. 7E, F).

Next, we determined agronomic traits of WT and *osa7* mutants. All parameters examined, including plant height, number of tillers per plant, number of panicles per plant, number of grains per plant, filling grain number per plant, 1000 grain weight, and yield, were significantly suppressed in *osa7* (*P*<0.05; Supplementary Table S1). The sucrose content of the flag leaf and seeds of the *osa7* mutant were decreased by ~36% and ~27%, respectively, compared with WT (Supplementary Fig. S8). In summary, we confirmed the importance of PM H^+^-ATPase for not only growth (Toda *et al.*, 2016), but also grain yield in rice.

### The physiological function of the flag leaf at the rice reproductive stage

**Fig. 8.**
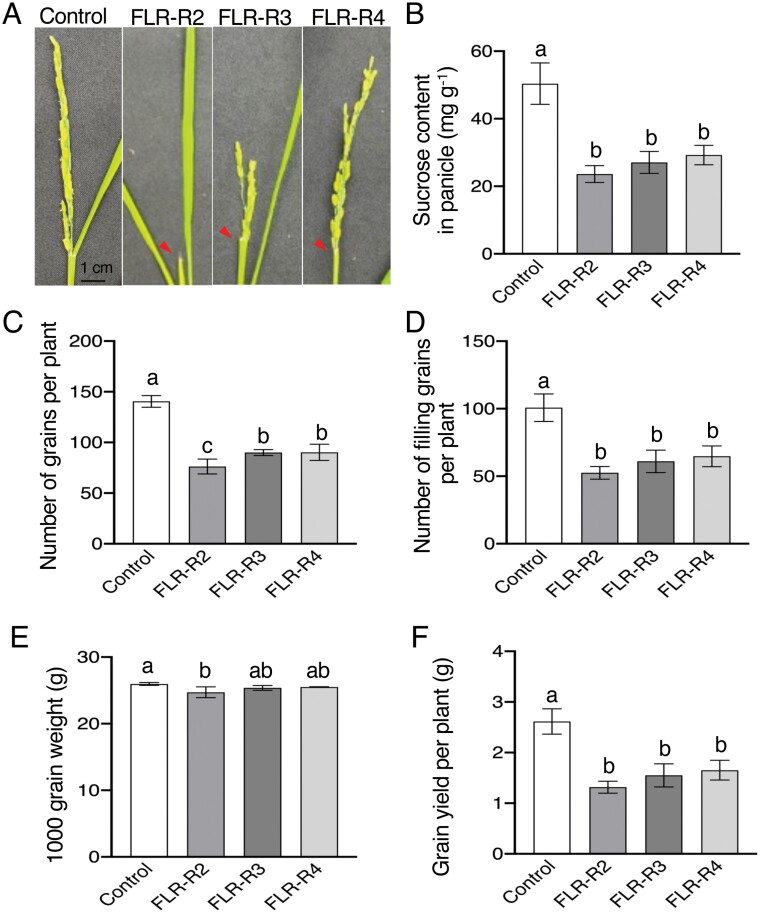
Characterization of flag leaf removal (FLR) treatment on grain yield. (A) Typical phenotypes of FLR plants at different growth stages (R2, flag leaf collar formation; R3, panicle exsertion from boot; R4, anthesis;) and control (no leaf removal). Red arrowheads indicate the positions of the flag leaf at Control, no leaf removal; R2, R3, and R4. (B) Sucrose content in panicles (mg g-1). (C) Number of grains per plant. (D) Number of filling grains per plant. (E) The 1000 grain weight. (F) Grain yield per plant. The data in (B)–(F) are means ±SD (*n*=4). Different letters indicate significant differences at *P*<0.001 according to a one-way ANOVA with Tukey’s multiple comparisons test.

Rice reproductive development consists of 10 growth stages from panicle initiation (R0) to complete panicle maturity (R9; Counce *et al.*, 2000). In this study, we investigated the contribution of the flag leaf to the accumulation of sucrose in panicles and agronomic traits at the flag leaf collar formation (R2), panicle exertion (R3), and anthesis (R4) stages by removing the flag leaf directly (Fig. 8A). Sucrose content in panicles of FLR plants at R2, R3, and R4 (FLR-R2, FLR-R3, and FLR-R4, respectively) was decreased by 53%, 46%, and 42%, respectively, compared with controls (Fig. 8B), which indicates that the flag leaf is important for carbohydrate accumulation in panicles during reproductive development. Similarly, agronomic traits, including the number of grains and filling grain number of FLR plants, were significantly reduced compared with controls (*P*<0.001; Fig. 8C, D). Grain yield decreased by approximately 49% in FLR-R2, 41% in FLR-R3, and 37% in FLR-R4 compared with controls (Fig. 8F). In summary, our data show that the flag leaf plays an important role in grain production by providing approximately 50% of the carbohydrate content.

## Discussion

Stomata play a vital role in plant growth and development by regulating the flow of gases in and out of plants, thereby directly impacting the photosynthetic capacity required for the supply of energy and carbohydrate in the grain (Jarvis and Morison, 1981; Jarvis and Davies, 1998; Jones, 1998). In this study, we found that cauline leaves exhibited enhanced stomatal density, stomatal index, and stomatal aperture, but slightly smaller stomatal size, than rosette leaves under similar growth conditions (Fig. 1). The higher stomatal index in cauline leaves (Fig. 1E) is similar to that in a previous report by Haus *et al.* (2018). These stomatal properties of cauline leaves contributed at least partially to enhanced photosynthetic activity (Fig. 2D). In addition, it has been demonstrated that an increased N content in leaves is correlated with a higher Rubisco activity level and photosynthetic rate (Li *et al.*, 2012). Therefore, we determined the Rubisco content and total N content. Our results show that both were significantly increased in cauline leaves compared with rosette leaves (*P*<0.001 for Rubisco, *P*<0.05 for N; Supplementary Fig. S9). Furthermore, we determined A-Ci curve and estimated the maximum Rubisco carboxylation rate (*V*_*cmax*_), the photosynthetic electron transport rate (*J*) and maximum rate of triose phosphate use (*TPU*) by fitting a photosynthetic model to the A-Ci curve (Supplementary Fig. S10). The cauline leaves had significantly higher *J*, *TUP* and *g*_*s*_ than rosette leaves by approximately 61%, 67%, and 25%, respectively (*P*<0.01 for *J* and *TPU*, *P*<0.05 for g_s_; Supplementary Fig. S10C–E). *V*_*cmax*_ also tends to be higher in cauline leaves, with no statistically significant difference compared with that of rosette leaves (*P*=0.069; Supplementary Fig. S10B), suggesting that Rubisco carboxylation rate is comparable between cauline and rosette leaves. Consequently, these results indicate that the increased photosynthetic activity in cauline leaves is due to higher stomatal conductance, increased N content associated with enhanced Rubisco content, and higher electron transport rate.

Light, such as blue light and red light, causes phosphorylation of the penultimate (Thr) residue of PM H^+^-ATPase in guard cells, thereby activating it and leading to stomatal opening (Kinoshita and Shimazaki, 1999; Ando and Kinoshita, 2018). In addition, up-regulation of PM H^+^-ATPase gene expression enhances light-induced stomatal opening in some cases (Wang *et al.*, 2014; Toh *et al.*, 2021; Zhang *et al.*, 2021). In this study, we found an increase of 12% in PM H^+^-ATPase protein expression in guard cells of cauline leaves compared with rosette leaves (Fig. 2A, B), and enhanced light-induced stomatal opening in cauline leaves (Fig. 2C; Supplementary Fig. S1). In contrast, the *AHA1* knockout mutant *aha1-9* (Yamauchi *et al.*, 2016) showed reduced PM H^+^-ATPase protein expression in guard cells of cauline leaves, and light-induced increase in stomatal conductance compared with WT (Fig. 3C). Furthermore, cauline leaves in *GC1::AHA2* exhibited enhanced guard cell PM H^+^-ATPase protein expression and light-induced stomatal opening compared with those in WT (Supplementary Fig. S2). Our results using cauline leaves from *aha1-9* mutant and *GC1::AHA2* plants are consistent with those from previous results using rosette leaves (Wang *et al.*, 2014; Yamauchi *et al.*, 2016). Therefore, guard cell PM H^+^-ATPase is a critical factor for enhanced light-induced stomatal opening and photosynthetic activity in cauline leaves (Supplementary Fig. S11).

It is still unknown why guard cells in cauline leaves show 12% higher PM H^+^-ATPase protein expression compared with rosette leaves (Fig. 2A, B). Our results also show that the expression level of *SOC1* in guard cell-enriched epidermis from cauline leaves is ~40% higher than that in rosette leaves (Fig. 4B; Supplementary Fig. S3B). *SOC1* has been suggested to increase PM H^+^-ATPase isoform expression (*AHA1*, *AHA2*, *AHA3*, *AHA5*, *AHA6*, *AHA10*, and *AHA11*) in guard cells (Kimura *et al.*, 2015; Aoki *et al.*, 2019).It is possible that the partially enhanced expression of *SOC1* in the guard cells of cauline leaves induces PM H^+^-ATPase expression. Further investigations are needed to clarify the molecular mechanism underlying the increased expression of guard cell PM H^+^-ATPase in cauline leaves compared with rosette leaves.

In canola, most of the carbon in the seeds comes from cauline leaves and stems rather than rosette leaves (Major and Charnetski, 1976). In Arabidopsis, cauline leaves are source organs at the reproductive stage, and regulate the partitioning of carbon and nitrogen organic matter to seeds (Takagi *et al.*, 2018). It has been proven that removal of the cauline leaves significantly reduces seed production in *Rumex crispus* (Maun and Cavers, 1971). This is consistent with the results of the present study, which indicates that CLR plants exhibited significantly decreased biomass and silique production compared with controls (Fig. 5), with no significant difference in silique weight. Similar effects were also observed in CLR experiments with the *aha1-9* mutant and *GC1::AHA2*-ox plants (Supplementary Figs S4, S6). Taken together, these results show that cauline leaves are important for seed production, as they provide photosynthetic products. In addition, transpiration in cauline leaves may affect seed production.

The flag leaf has higher photosynthetic activity and stomatal conductance than other leaves in rice plants (Zhang *et al.*, 2005), and in barley, stomatal density is higher in flag leaf than in other leaves (Yoshida, 1977). However, leaf age was not taken into account in these studies. Therefore, we conducted experiments using the flag leaf and second youngest leaf of the same age (Fig. 6), and found that the flag leaf actually had higher stomatal conductance and photosynthetic activity than the second youngest leaf. In addition, we found that the stomata were similar in size but had a higher density than those in the second youngest leaf, and the chlorophyll content was higher in the flag leaf than in the second youngest leaf (Fig. 6B–D). These results suggest that the higher photosynthetic activity in the flag leaf is due to higher stomatal conductance, stomatal density, and at least in part, chlorophyll content. Further investigation will be needed to determine nitrogen and Rubisco content, because these are known to be strongly related to photosynthetic capacity in rice (Makino *et al.*, 1997, Makino *et al.*, 2000). To the best of our knowledge, these observations represent a detailed evidence regarding the stomatal properties of the flag leaf in rice.

The PM H^+^-ATPase is highly expressed in the guard cells of plants, including rice and Arabidopsis (Toda *et al.*, 2016; Inoue and Kinoshita, 2017). Therefore, it is likely that guard cell PM H^+^-ATPase in the flag leaf contributes to higher stomatal conductance. Indeed, the flag leaf of *osa7-1* showed no differences in stomatal size or density, but lower stomatal conductance and photosynthetic activity compared with WT (Fig. 7C–F), which was associated with a reduced sucrose content in the flag leaf and seeds (Supplementary Fig. S8). Furthermore, a previous study showed that up-regulation of PM H^+^-ATPase gene ­expression markedly improves light-induced stomatal opening, plant growth, and grain yield in rice (Zhang *et al.*, 2021). Taken together, these results suggest that PM H^+^-ATPase is also critical for the stomatal properties of the flag leaf and grain yield. In addition, previous studies showed that the insertion angle of the flag leaf differs among rice varieties, which affects the photosynthetic activity (Kobayashi *et al.*, 2003; Jiang *et al.*, 2022). Furthermore, cytokinin was suggested to increase the stomatal density in tomato and make a great contribution to leaf morphogenesis (Farber *et al.*, 2016; Muszynski *et al.*, 2020). It is possible that these genetic factors and mechanisms also contribute to the improved stomatal properties and photosynthetic activity of the flag leaf; this should be investigated further.

It has been demonstrated that the flag leaf makes a contribution of more than 18% to the grain yields in rice (Abou-Khalifa *et al.*, 2008; Fatima *et al.*, 2019). However, no detailed data have been reported regarding the effects of the flag leaf on grain yield at different stages of panicle growth. In this study, by removing the flag leaf at three growth stages of rice (R2, R3, and R4; Fig. 8A), we found that removal of the flag leaf at R2 significantly reduced the grain yield (~50%) and sucrose content (53%) in panicles (Fig. 8B–F). This is consistent with previous reports suggesting that the flag leaf contributed to approximately 50% of the assimilates for grain filling in rice (Yoshida, 1981; Ishii, 1993; Nakano *et al.*, 1995; Li *et al.*, 1998; Adachi *et al.*, 2017). These results indicate that the flag leaf has the greatest impact on grain yield in the early stage of grain filling.

In summary, our study illustrates specific stomatal properties in the cauline leaves of Arabidopsis and the flag leaf of rice. PM H^+^-ATPase plays a crucial role in regulating stomatal aperture, photosynthesis, seed production, and grain yield in cauline leaves and the flag leaf. These findings provide a deeper understanding of the characteristics of cauline leaves in the dicot Arabidopsis and the flag leaf in the monocot rice.

## Supplementary data

The following supplementary data are available at *JXB* online.

Fig. S1. Stomatal apertures of Col-0 rosette and cauline leaves in response to light and ABA treatment.

Fig. S2. Stomatal properties of cauline leaves of *GC1::AHA2* transgenic plants.

Fig. S3. qRT–PCR assay for *AHA2* and *SOC1* gene expression.

Fig. S4. Productivity of *aha1-9* mutants.

Fig. S5. Sucrose content of cauline leaves of *aha1-9* mutants.

Fig. S6. Seed production following cauline leaf removal (CLR) in Col-0, empty vector transformant (EV), and *GC1::AHA2* transgenic plants.

Fig. S7. Epidermal peels of the flag leaf from rice at the reproductive stage and fully expanded second leaf from rice at the vegetative stage stained with ruthenium red.

Fig. S8. Sucrose content in the flag leaf and panicles of WT plants and *osa7-1* mutants.

Fig. S9. Nitrogen and Rubisco content in cauline and rosette leaves from 6-week-old Col-0 plants.

Fig. S10. Photosynthesis in cauline and rosette leaves from 6-week-old Col-0 plants.

Fig. S11. Schematic model of PM H^+^-ATPase function in stomatal movement and photosynthesis in cauline and rosette leaves of Col-0.

Table S1. Agronomic traits and grain yields of *osa7-1* mutant plants.

erac492_suppl_supplementary_figures_S1-S11_table_S1

## Data Availability

All data supporting the findings of this study are available within the paper and its supplementary material published online.
