# Supplementary material for: Stomatal properties of Arabidopsis cauline and rice flag leaves and their contributions to seed production and grain yield
Source: J Exp Bot. 2022 Dec 15;74(6):1957–73. doi: 10.1093/jxb/erac492 (PMC10049919; doi:10.1093/jxb/erac492)
Supplement: erac492_suppl_supplementary_figures_S1-S11_table_S1 [file erac492_suppl_supplementary_figures_s1-s11_table_s1.pdf]

Supplementary figures

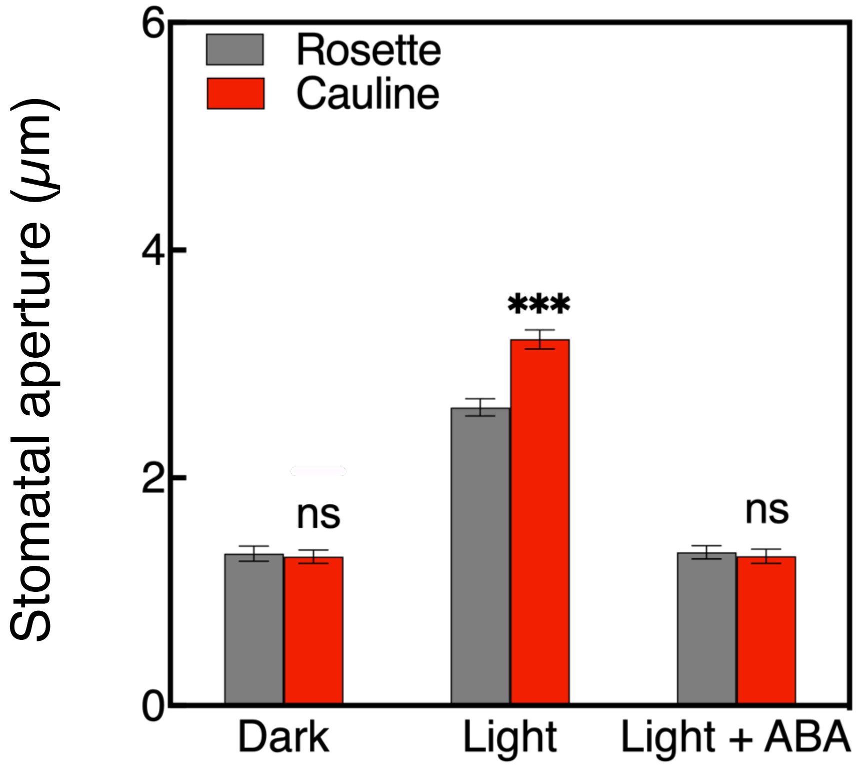

Supplementary Fig. S1. Stomatal apertures of Col-0 rosette and cauline leaves in response to light and ABA treatment. Epidermal tissue from dark-adapted plants in the stomatal opening buffer was kept in the dark for 2.5 h or illuminated with light (10 μmol m^{-2} s^{-1} blue light superimposed on 50 μmol m^{-2} s^{-1} red light) for 2.5 h or light in the presence of 20 μM ABA. Data are means ± SD ($n = 3$) of 40 stomata in each sample. Asterisks indicate significant differences from corresponding rosette leaves (Student's *t* test; ***$P < 0.001$; ns, not significant).

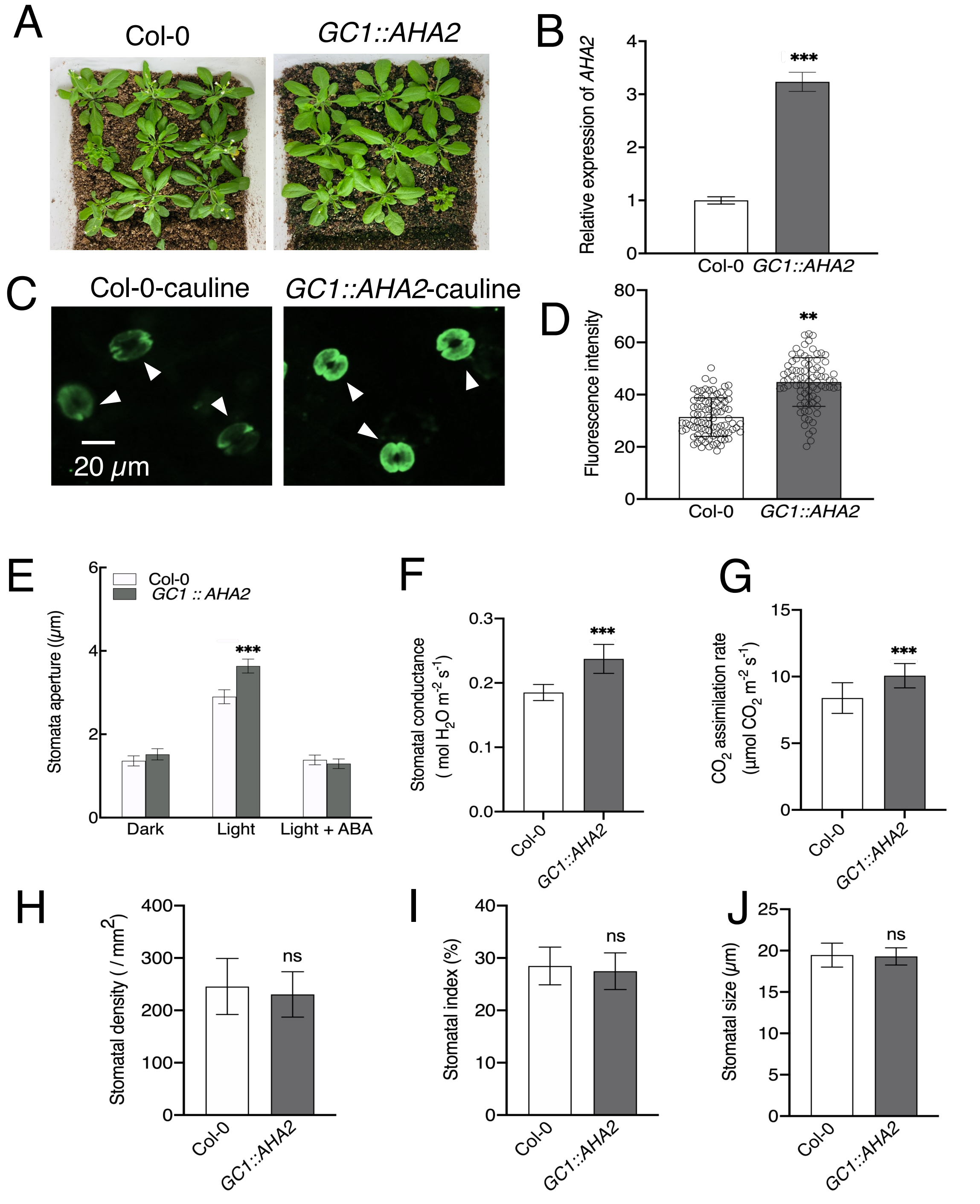

Supplementary Fig. S2. Stomatal properties of cauline leaves of *GC1::AHA2* transgenic plants. (A) Phenotypes of 25-day-old Col-0 and *GC1::AHA2* transgenic plants grown under conditions of high light illumination (200 μmol m^{-2} s^{-1}) in a growth room. (B) qRT-PCR assay of *AHA2* gene expression in guard cell–enriched epidermal fragments of cauline leaves from Col-0 and *GC1::AHA2* transgenic plants. Data are means ± SD ($n = 3$) of three plants in each experiment. Gene expression was calculated relative to *β-TUB2*. Asterisks indicate significant differences from the corresponding Col-0 (Student's *t* test; ***$P < 0.001$). (C) Typical fluorescence images of PM H^+-ATPase in guard cells of the epidermis from cauline leaves of Col-0 and *GC1::AHA2* transgenic plants (for detailed conditions of the immunohistochemical assay, see Materials and Methods). White arrowheads indicate positions of stomata in the epidermis. (D) Quantification of fluorescence intensities of PM H^+-ATPase in guard cells corresponding to (C). Data are means ± SD ($n = 3$) of 30 stomata in each experiment. Asterisks indicate significant differences from the corresponding Col-0 (Student's *t* test; **$P < 0.01$). (E) Stomatal opening in response to light or light with 20 μmol ABA in cauline leaves of Col-0 and *GC1::AHA2* transgenic plants. Other details are the same as in Supplementary Fig. S1. Asterisks indicate significant differences from the corresponding Col-0 (Student's *t* test; ***$P < 0.001$). Stomatal conductance (F) and CO_2 assimilation rate (G) of cauline leaves of Col-0 and *GC1::AHA2* transgenic plants. Measurements were conducted under white light of 750 μmol m^{-2} s^{-1}. Other details are the same as in Fig. 2C, D. Asterisks indicate significant differences from the corresponding Col-0 (Student's *t* test; ***$P < 0.001$). (H) Stomatal density of Col-0 and *GC1::AHA2* transgenic plants cauline leaves. Values represent means ± SD ($n = 3$) of 9-10 leaves in each experiment. (I) Stomatal index of Col-0 and *GC1::AHA2* transgenic plants cauline leaves. Data are means ± SD ($n = 3$) of 9-10 leaves in each experiment. (J) Stomatal size of Col-0 and *GC1::AHA2* transgenic plants cauline leaves. Data are means ± SD ($n = 3$) of 40-50 stomata in each experiment.

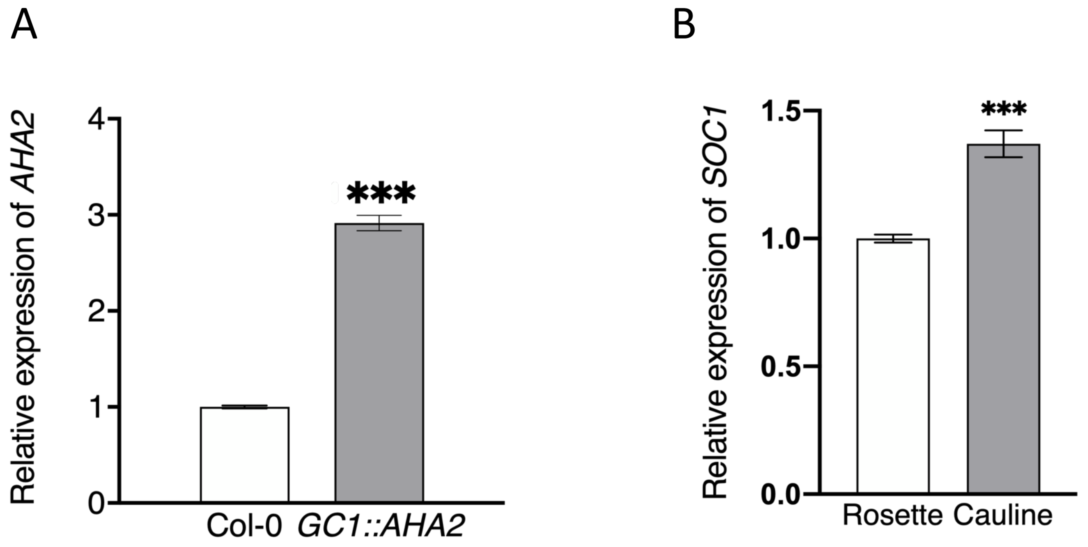

Supplementary Fig. S3 qRT– PCR assay for *AHA2* and *SOC1* gene expression. (A) Relative expression level of *AHA2* gene in guard cell–enriched epidermal fragments of cauline leaves from Col-0 and *GC1::AHA2*. Data are means ± SD (*n* = 3). Gene expression was calculated relative to *UBQ5.* Asterisks indicate significant differences from the corresponding Col-0 plants (Student's *t* test; ****P* < 0.001). (B) Relative expression level of *SOC1* in guard cell-enriched fragments of rosette and cauline leaves from Col-0 plants. Data are means ± SD (*n* = 8). Gene expression was calculated relative to *UBQ5.* Asterisks indicate significant differences from the corresponding rosette leaves (Student's *t* test; ****P* < 0.001).

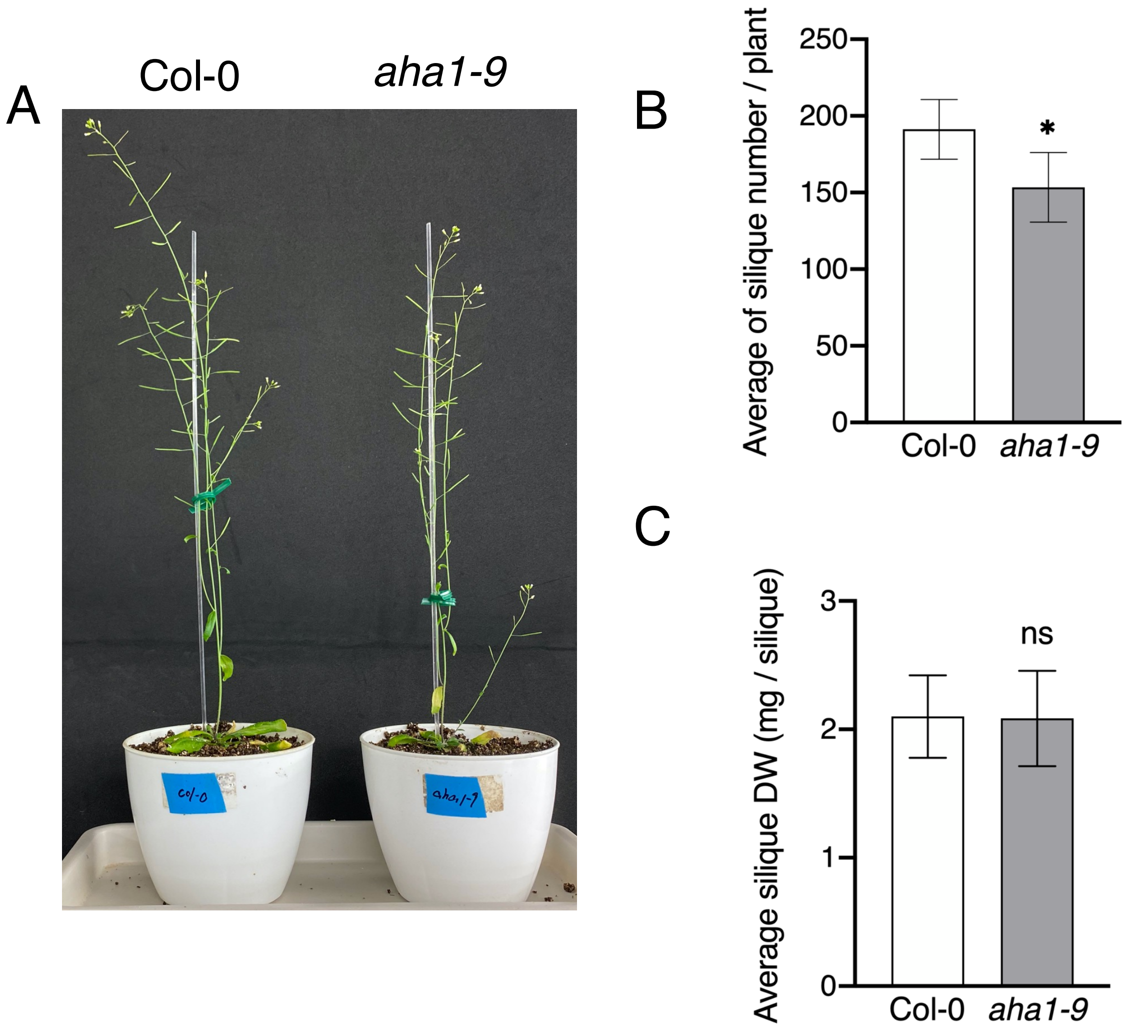

Supplementary Fig. S4. Productivity of *aha1-9* mutants. (A) Typical phenotypes of Col-0 and *aha1-9* mutants grown under white light (150 µmol m^{-2} s^{-1}) for 7 weeks in a growth room. (B) Average number of siliques per plant. Data are means ± SD ($n = 6$). Asterisks indicate significant differences from the corresponding Col-0 (Student's *t* test; $*P < 0.05$). (C) Average silique dry weight per plant. Data are means ± SD ($n = 6$). Differences from the corresponding Col-0 were examined for significance with Student's *t* test (ns, not significant).

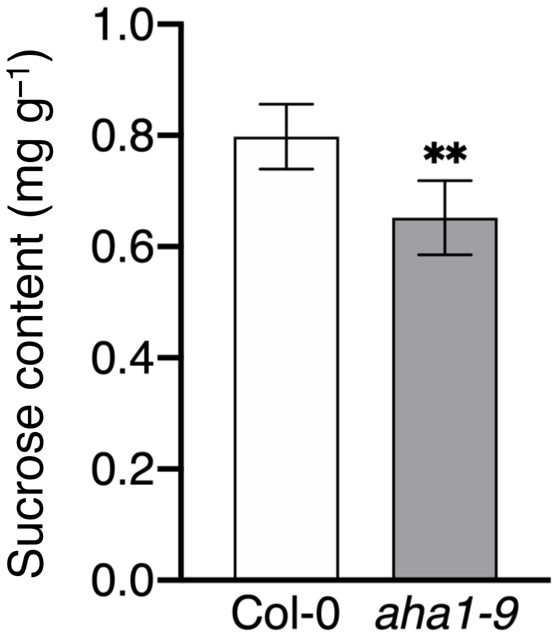

Supplementary Fig. S5. Sucrose content of cauline leaves of *aha1-9* mutants. Sucrose content of cauline leaves of Col-0 and *aha1-9* mutants were measured as described in Materials and Methods. Data are means ± SD ($n = 3$) of five plants in each experiment. Asterisks indicate significant differences from the corresponding Col-0 (Student's t test; $^{**}P < 0.01$).

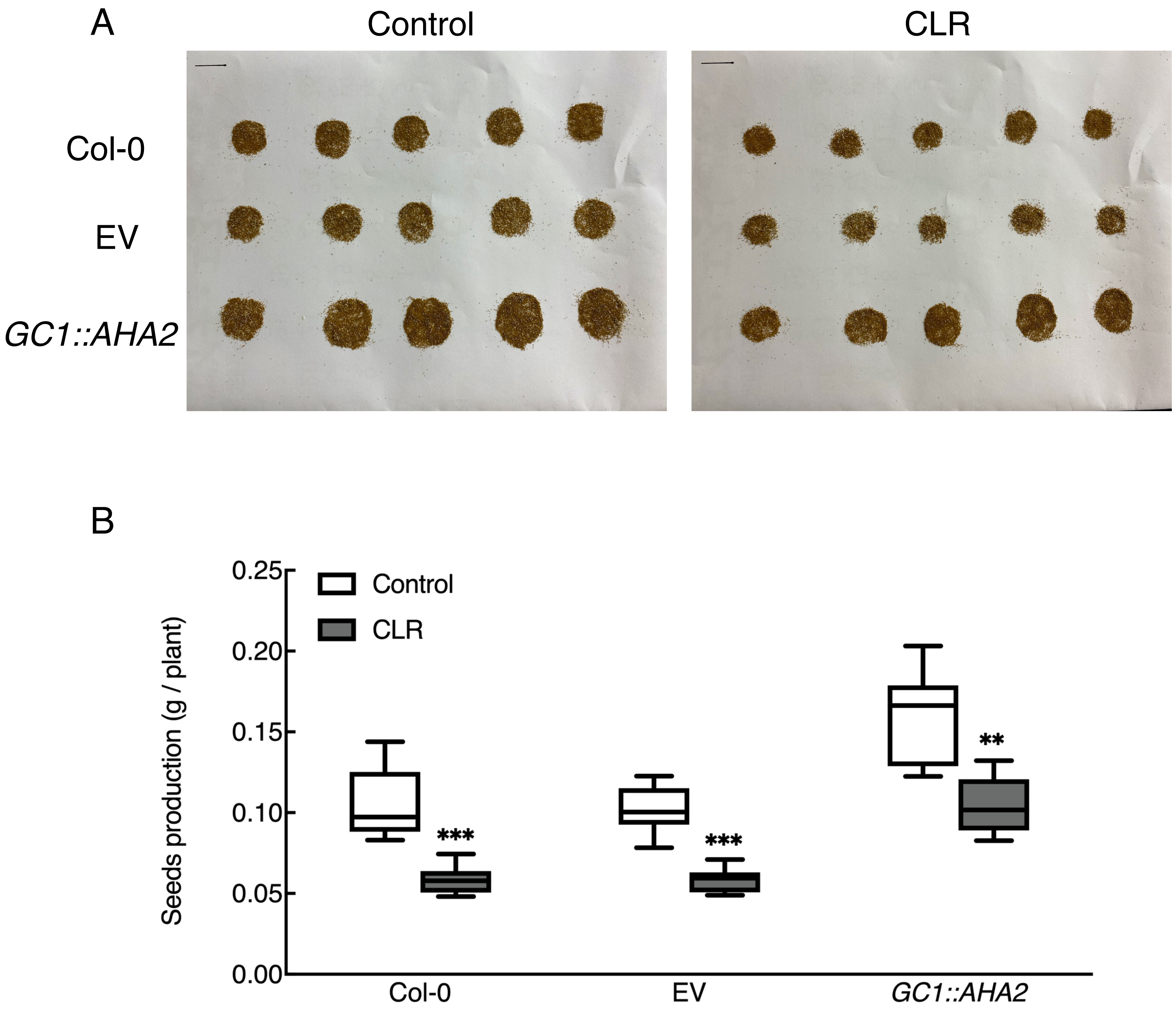

Supplementary Fig. S6. Seed production following cauline leaf removal (CLR) in Col-0, empty vector transformant (EV), and *GC1::AHA2* transgenic plants. (A) Images of seeds from five individual Col-0, EV, and *GC1::AHA2* transgenic plants with or without CLR treatment. (B) Seed production of Col-0, EV, and *GC1::AHA2* transgenic plants with or without CLR treatment. Values represent means ± SD ($n = 9$). Asterisks indicate statistically significant differences from the corresponding control (Student's t test; $^{**}P < 0.01$).

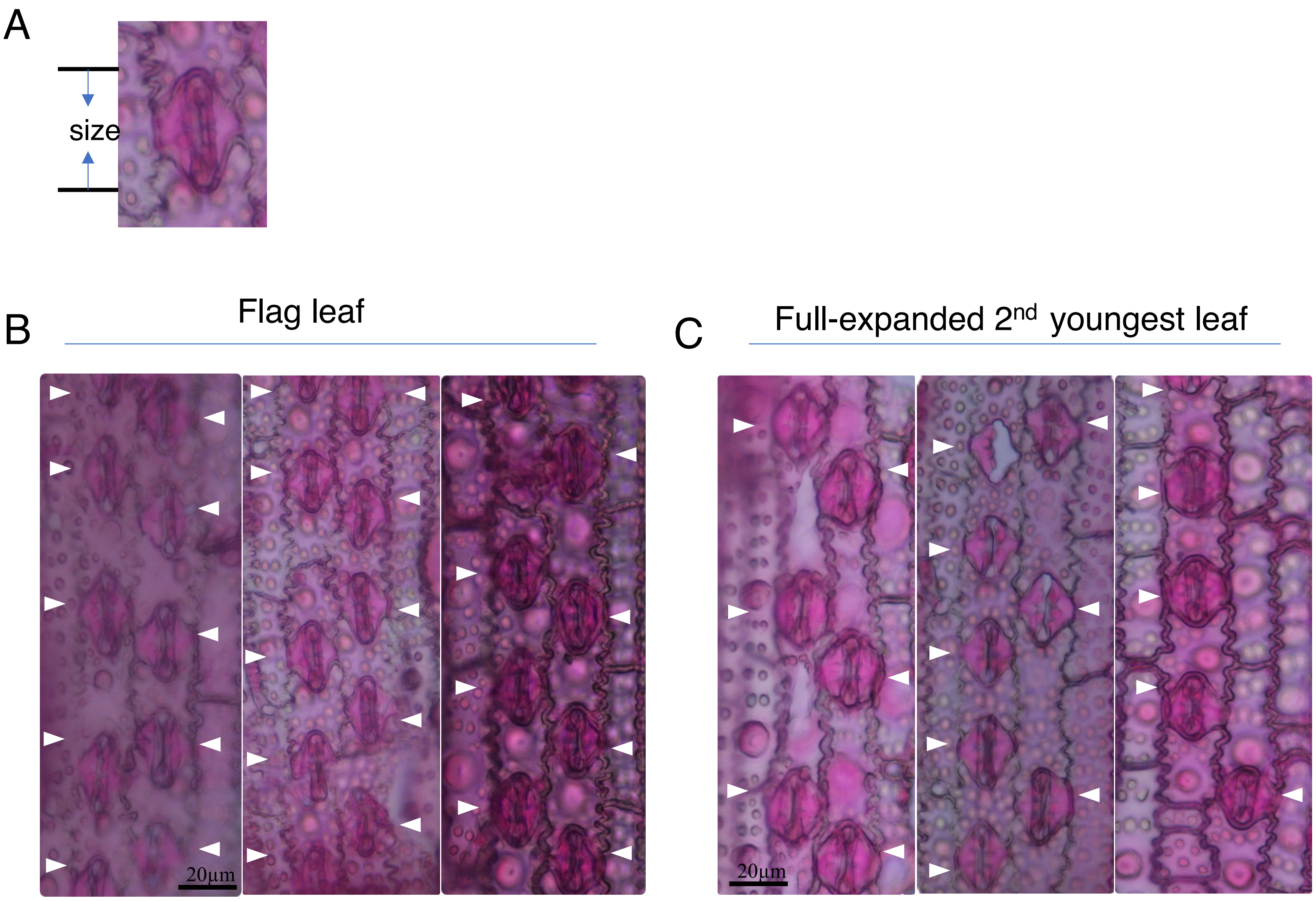

Supplementary Fig. S7. Epidermal peels of the flag leaf from rice at the reproductive stage and fully expanded second youngest leaf from rice at the vegetative stage stained with ruthenium red. (A) Schematics indicate the stomatal size of rice leaf blades with blue arrows. Phenotypes of stomata after ruthenium red staining in the flag leaf of rice (B) at the reproductive stage and fully expanded second youngest leaf of rice (C) at the vegetative stage (for details of ruthenium red staining procedure, see the Materials and Methods). Scale bars = 20 µm. White arrowheads indicate the positions of stomata in each leaf.

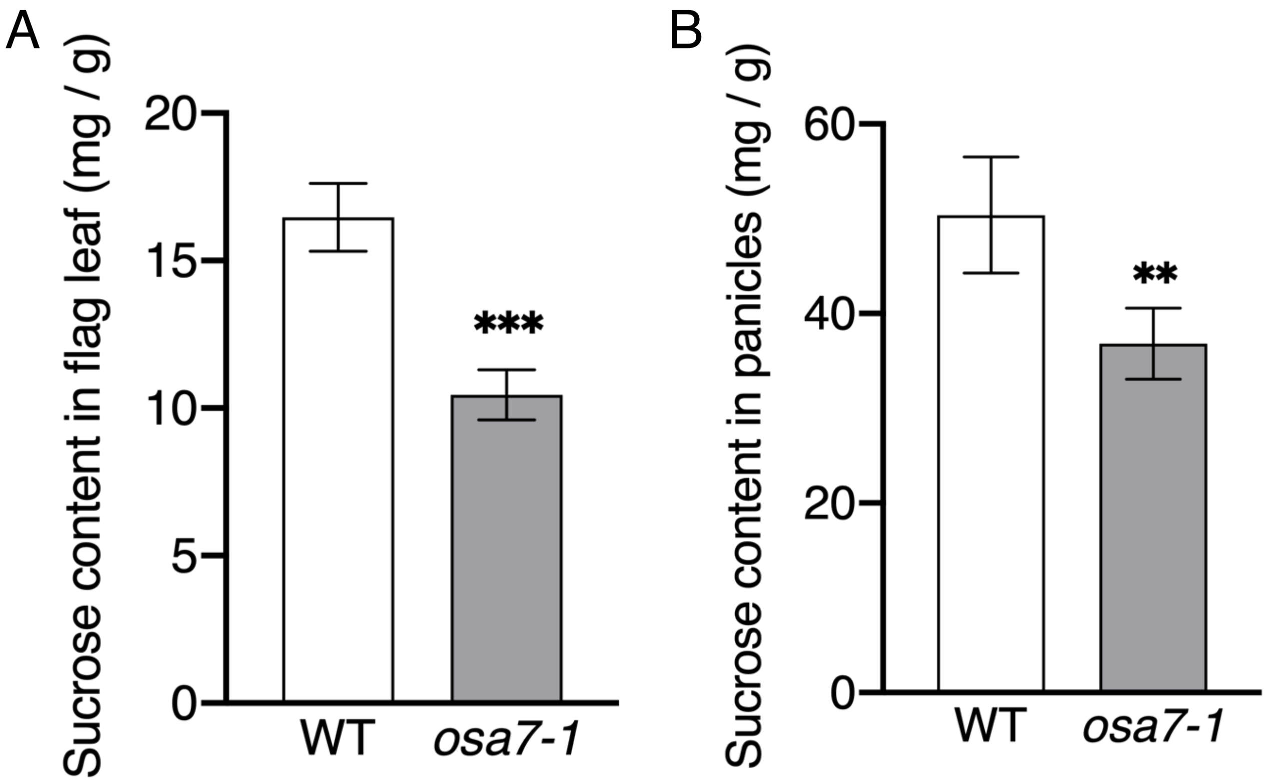

Supplementary Fig. S8. Sucrose content in the flag leaf and panicles of WT plants and *osa7-1* mutants. (A) Sucrose content in the flag leaf of WT and *osa7-1* mutants. Data are means ± SD ($n = 4$). Asterisks indicate significant differences from the corresponding WT (Student's t test; ***$P < 0.001$). (B) Sucrose content in the panicles of WT and *osa7-1* mutants. Data are means ± SD ($n = 4$). Asterisks indicate significant differences from the corresponding WT (Student's t test; **$P < 0.01$).

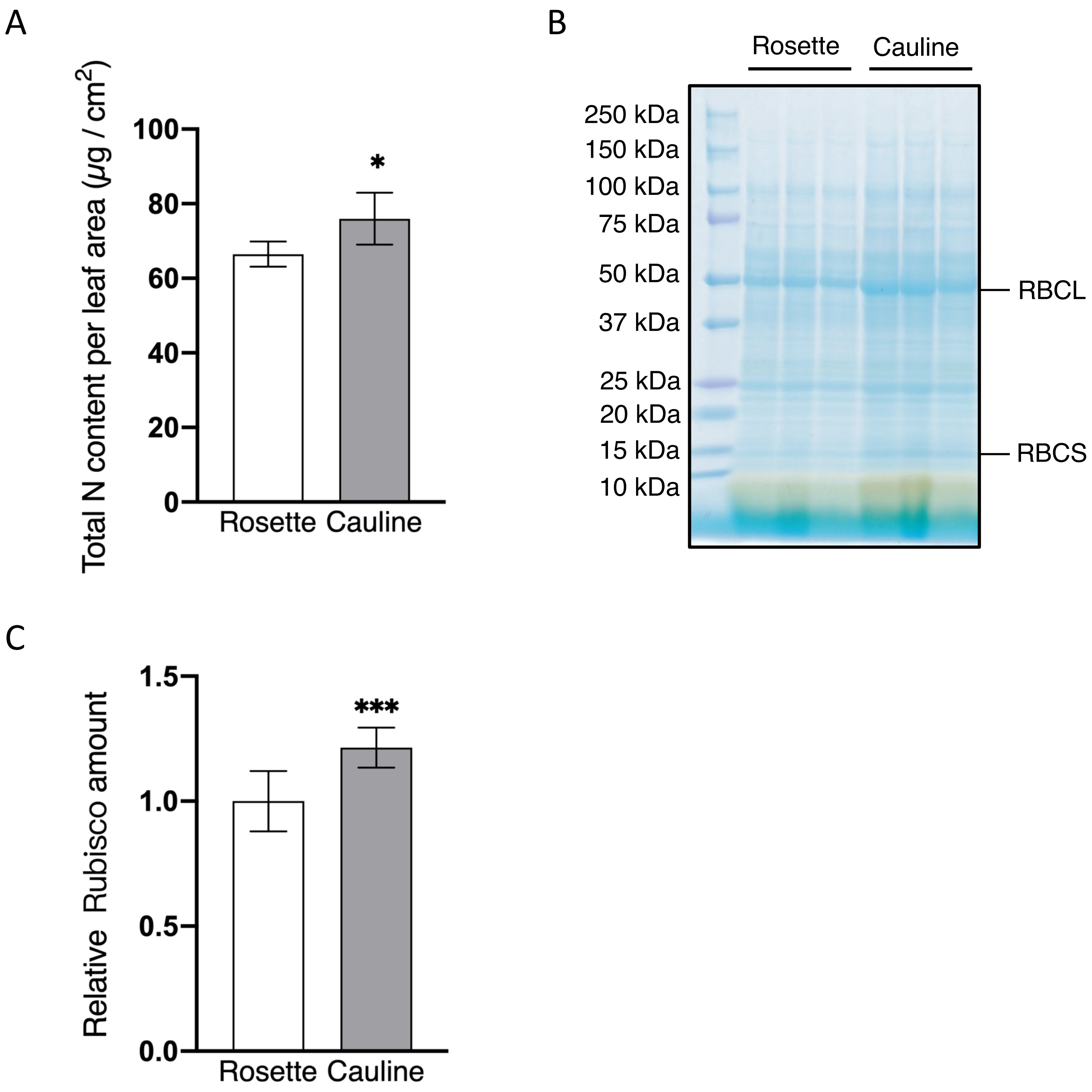

Supplementary Fig. S9. Nitrogen and Rubisco content in cauline and rosette leaves from 6-week-old Col-0 plants. (A) Total nitrogen content per leaf area in cauline and rosette leaves. Data are means ± SD ($n = 6$). Asterisks indicate significant differences from the corresponding rosette leaves (Student's *t* test; ***$P < 0.001$). (B) SDS-PAGE analysis for Rubisco content determination by CBB staining. Three independent rosette and cauline leaves (4 mg) were homogenized and centrifuged (for details, see the Materials and Methods) to extract soluble proteins and an equivalent volume of the supernatant from each sample was separated by SDS-PAGE. Three biological replicates were performed. RBCL, Rubisco large subunit; RBCS, Rubisco small subunit. (C) The Rubisco amount in cauline leaves was calculated by ImageJ software and normalized relative to that of rosette leaves given an arbitrary value of 1. Data are means ± SD ($n = 9$). Asterisks indicate significant differences from the corresponding rosette leaves (Student's *t* test; ***$P < 0.001$). Total Rubisco content included RBCL and RBCS.

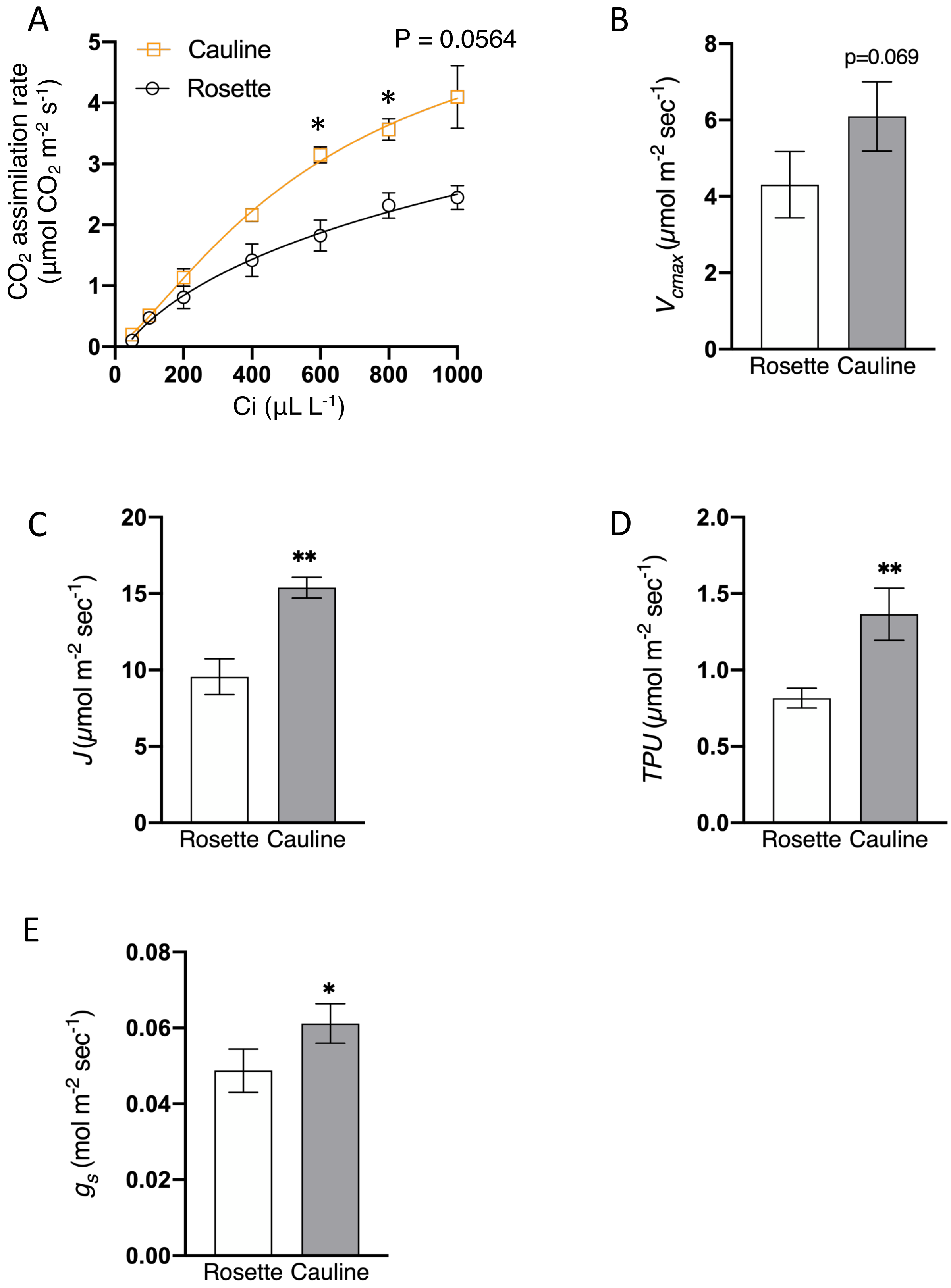

Supplementary Fig. S10. Photosynthesis in cauline and rosette leaves from 6-week-old Col-0 plants. (A) The responses of leaf CO_2 assimilation to intercellular CO_2 concentration (Ci) under saturated light (750 µmol m^{-2} s^{-1}) condition in cauline and rosette leaves. Data are means ± SD (n=3). Asterisks indicate significant differences from the corresponding rosette leaves (Student's *t* test; **P* < 0.05). (B-D) Maximum Rubisco carboxylation rate (V_{cmax}), photosynthetic electron transport rate (*J*) and maximum rate of triose phosphate use (*TPU*) fitted to the A–Ci curves. Data are means ± SD (n=3). Asterisks indicate significant differences from the corresponding rosette leaves (Student's *t* test; ** *P* < 0.01). (E) Stomatal conductance (g_s) obtained from data points collected at 400 µL L^{-1} CO_2. Data are means ± SD (n=3). Asterisks indicate significant differences from the corresponding rosette leaves (Student's *t* test; **P* < 0.05).

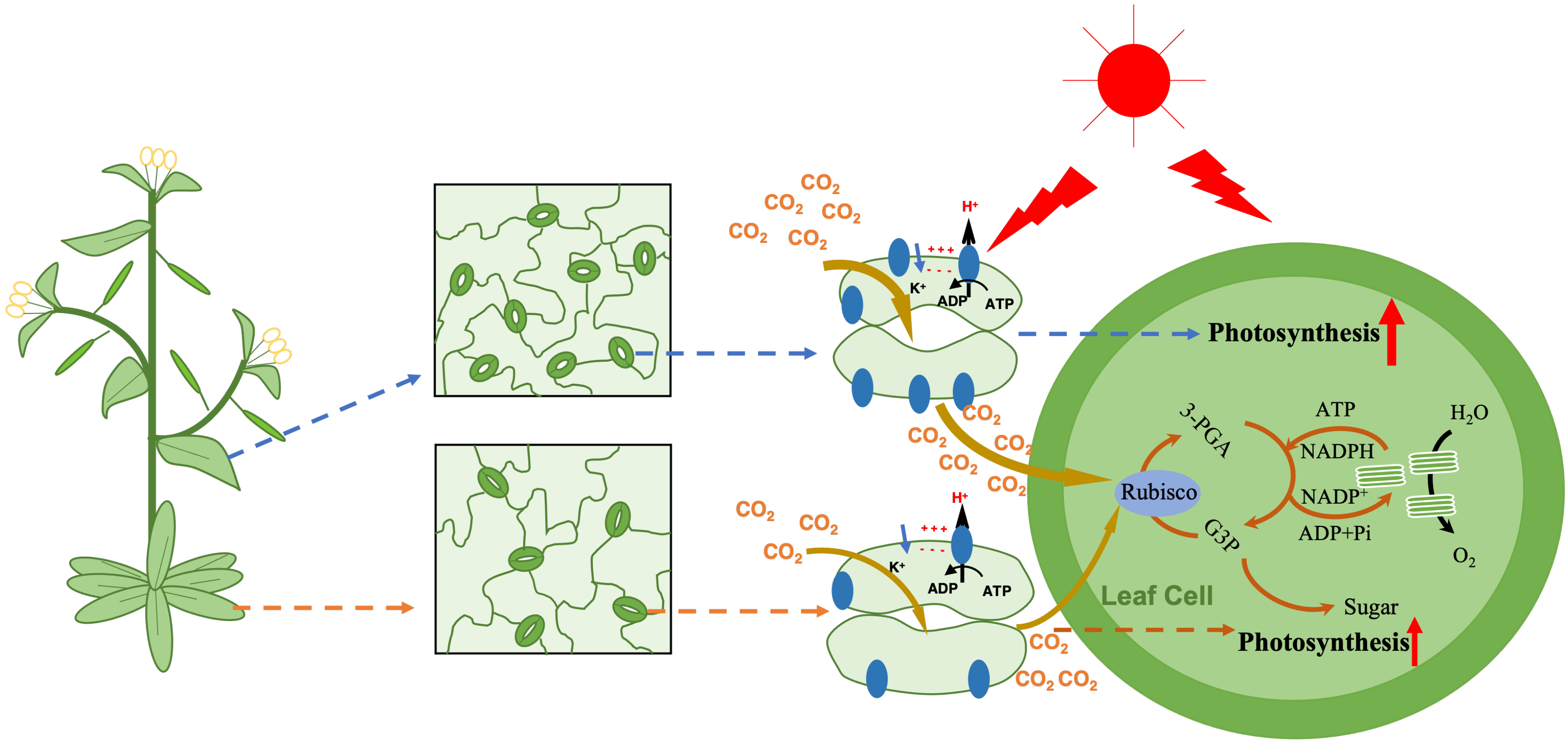

Supplementary Fig. S11. Schematic model of PM H$^+$-ATPase function in stomatal movement and photosynthesis in cauline and rosette leaves of Col-0. The stomatal density and index of cauline leaves were much higher than those of rosette leaves. PM H$^+$-ATPase in guard cells of cauline leaves polarised the membrane potential, thus allowing K$^+$ influx for the opening of stomata under light conditions. Cauline leaves showed enhanced stomatal opening and photosynthetic activity compared to rosette leaves due to the increased PM H$^+$-ATPase level. Blue circles indicate PM H$^+$-ATPase in guard cells. G3P, glyceraldehyde 3-phosphate; PGA, phosphoglycerate; RuBisCO, ribulose-1,5-bisphosphate carboxylase.

Supplementary Table S1. Agronomic traits and grain yields of *osa7-1* mutant plants. Plant height, number of tillers, number of panicles, number of grains, filling grain number, 1000 grain weight, and grain yield of WT and *osa7-1* mutants. Data are means ± SD (n = 4). Asterisks indicate significant difference from the corresponding WT plants (Student's t test; *, $P < 0.05$).

Cultivar	Plant height (cm)	Tiller number per plant	Panicle number per plant	number of grains per plant	number of filling grains per plant	1000 grain weight (g)	grain yield per plant (mg / g)
WT	75±2.5	14.8±2.2	10.2±1.7	140.5±5.8	100.2±3.4	26±0.21	2.6±0.25
osa7-1	54±4.4*	9.25±1.2*	5.8±1.3*	77±1.8*	53.3±4.1*	25.1±0.47*	1.3±0.19*
